# Plant responses to gaseous pollutants, biochemical and transcriptomic insights

**DOI:** 10.3389/fpls.2026.1768073

**Published:** 2026-03-16

**Authors:** Malik Urfa Gul, M. Junaid Gul, Muhammad Hafiz Raza Ur Rehman, Gyu Sang Choi, Chang-Hyeon Park

**Affiliations:** IT Department School of Computer Science and Engineering, Yeungnam University, Gyeongsan, Gyeongbuk, Republic of Korea

**Keywords:** abiotic stress and crop resilience, and stomatal regulation, atmospheric gaseous pollutants, photosynthesis, plant biochemical stress responses, pollutant plant interactions, reactive oxygen and nitrogen species (ROS, RNS), respiration

## Abstract

Atmospheric gaseous pollutants sulfur dioxide (SO_2_), nitrogen oxides (NO_x_), ozone (O_3_), and carbon monoxide (CO) increasingly co-occur in crop canopies and cause damage that spans atmospheric chemistry, redox signaling, and whole-leaf function. Prior work is often fragmented by single pollutant, single endpoints, or single scale, which limits mechanistic comparability and makes it difficult to build computationally useful models that generalize across environments. This synthesis integrates the atmospheric-to-cellular continuum in a form tended for quantitative plant science and computational researchers. We connect pollutant formation and microclimate-driven exposure to stomatal uptake, apoplastic speciation, subcellular targets, and downstream impacts on photosynthesis, respiration, and stomatal regulation. At the biochemical level, we unify key reaction routes and control points, SO_2_ hydration to bisulfite and sulfite and the associated detoxification demands, NO_x_ driven redox interconversion and nitrosative stress with protein modification, O_3_ decomposition to reactive oxygen species (ROS) and membrane/chloroplast injury with guard-cell dysfunction. We also clarify the agronomic relevance of CO as a heme-centered modifier that can reshape respiration-linked redox balance and stress signaling, particularly under multi-pollutant mixtures. Beyond summarizing mechanisms, our novelty in this synthesis, is not to repeat well-known single-gas mechanisms, but to bring together key studies that are rarely discussed side by side and show how their results can be used in a practical, quantitative way. Specifically, we organize evidence across SO_2_, NO_x_, O_3_, and CO around shared convergence nodes (ROS and RNS buffering, antioxidant cycling, and electron-transport constraints), and we translate those mechanisms into discriminative mechanistic fingerprints that can be treated as measurable biomarkers or model features. To support translation, we summarize how prior studies typically quantify dose and outcomes using open-top chambers, FACE, and flux-based datasets that connect stomatal uptake to redox status and yield-related traits. This enables more consistent dataset design, model constraints for machine learning, and interpretable prediction of tolerance and yield risk under realistic multi-pollutant atmospheres.

## 1 Introduction

Over the past century, anthropogenic activities such as industrialization, vehicular emissions, power generation, and urbanization have profoundly altered the composition of Earth’s atmosphere (Hale, 1993). Among the most pervasive changes is the increased concentration of reactive gaseous pollutants, particularly sulfur dioxide (SO_2_), nitrogen oxides (NO_x_), ozone (O_3_), and carbon monoxide (CO) (Meo and Ayoub, 2024). These gases, emitted through combustion processes, fossil fuel use, and industrial exhausts, are not only central to atmospheric chemistry but also potent agents of biological disruption in terrestrial ecosystems (Odubo et al., 2024). Plants, being the primary autotrophs and dominant surface interface in terrestrial biomes, are chronically exposed to these gaseous toxins through stomatal uptake and surface adsorption (Landmeyer, 2012). While the broader ecological impacts of air pollution are widely acknowledged, the biochemical mechanisms by which these atmospheric gases interfere with core physiological processes such as respiration, photosynthesis, and stomatal function remain underexamined in an integrated framework (Husen, 2021).

The biochemical consequences of pollutant exposure in plants are multifaceted and highly reactive. For instance, SO_2_, after entering the leaf apoplast, is hydrated to form bisulfite (HSO_3_^-^) and sulfite (SO_3_²^-^), which are strong nucleophiles capable of reacting with chlorophyll, DNA bases, and thiol-containing proteins (Singh and Lamabam, 2012; Majumdar, 2023). These reactions lead to degradation of chloroplast pigments, inhibition of enzymatic function, and altered redox signaling (Brychkova, 2007). NO_x_ gases, particularly NO and NO_2_, enter the mesophyll and participate in redox cycling, producing reactive nitrogen species (RNS) such as peroxynitrite (ONOO^-^), which induce oxidative and nitrosative stress (Corpas and Barroso, 2013a). Ozone (O_3_), a secondary pollutant formed via photochemical reactions involving NO_x_ and VOCs, is perhaps the most phytotoxic gas due to its high oxidative potential (Monks, 2015). Upon entry through stomata, O_3_ decomposes to singlet oxygen and superoxide radicals, which damage cellular membranes, photosystem II proteins, and metabolic enzymes. Even CO, though relatively less reactive in air, interferes with heme-containing respiratory enzymes by competitively binding to cytochrome oxidase, impeding electron transport in both mitochondria and chloroplasts (Pellegrini, 2011; Muneer, 2014). CO is included not as a generic “damage gas,” but because its heme affinity provides a plausible route to constrain respiration-linked electron transport and to modulate stress signaling, particularly when oxidative or nitrosative load is already elevated.

The implications of these pollutant-induced disruptions are far-reaching. At the cellular level, damage to chloroplasts impairs carbon fixation and energy metabolism. At the tissue level, changes in stomatal aperture and dynamic transpiration affect water use efficiency and nutrient uptake (Ran, 2017). These physiological stress responses cascade into reduced plant productivity, altered phenology, and increased vulnerability to pathogens. Importantly, such stress syndromes are not confined to agricultural crops alone; forest trees, ornamental plants, and natural vegetation are equally susceptible, threatening both biodiversity and ecosystem services (Duque et al., 2021; Cheesman, 2024). Furthermore, since plants serve as the foundational trophic level in most food webs, their compromised function under air pollution not only jeopardizes food security but also influences the global carbon cycle and climate feedback mechanisms (Zhu et al., 2022; Mao et al., 2024). What is missing, and what the title implies, is an omics-grounded synthesis. Many pollutant studies now report transcriptomic responses, yet the gene-level outputs are typically presented as long lists of differentially expressed genes with limited cross-pollutant mapping. Without linking transcriptional programs to shared biochemical state variables (redox buffering, sulfur assimilation demand, electron-transport constraint, guard-cell regulation), transcriptomic results remain descriptive and cannot reliably explain why mixtures behave differently from single gases. In practice, pollutant tolerance is often controlled by the interaction between early redox chemistry and downstream gene regulation, including antioxidant reprogramming, sulfur and nitrogen metabolism, hormone and MAPK signaling, and stomatal regulatory modules. This is the exact point where transcriptomic insights should strengthen mechanistic inference, but current reviews rarely organize the evidence in a way that makes this connection explicit across gases.

Recent studies have increasingly highlighted the biochemical dimension of air pollution effects on vegetation. For example, researchers demonstrated that elevated tropospheric ozone concentrations significantly suppress Rubisco activity and photosynthetic rate in wheat cultivars (Feng, 2024). Similarly, A study provided a mechanistic overview of how NO_2_ alters antioxidant enzyme dynamics in crop plants, contributing to premature leaf senescence (Liu et al., 2015). Other investigations have shown that SO_2_ exposure modulates sulfur metabolism, leading to accumulation of non-protein thiols like glutathione as part of a detoxification response. These studies underscore that pollutant-plant interactions are governed by a reactive coupling between pollutant chemistry and plant redox and regulatory networks, rather than being explained only as a generic physical stress (Yarmolinsky et al., 2013; Li et al., 2022).

Despite this, most research remains compartmentalized, focused either on specific pollutants or isolated plant responses. What remains lacking is a comprehensive survey that maps the full chemical reactivity of major gaseous pollutants across plant physiological systems (Concas et al., 2021; Custer and Dini-Andreote, 2022; Ossola and Farmer, 2024; Upadhayay et al., 2024). This review addresses that gap by synthesizing recent findings on pollutant chemistry, reaction mechanisms inside plant tissues, and their downstream physiological effects. In doing so, it contributes to a deeper understanding of plant resilience, the biochemical basis of pollutant toxicity, and potential strategies for developing pollution-tolerant crops.

Given the rising levels of air pollution globally, particularly in rapidly urbanizing regions, and the accelerating climate crisis, understanding how gaseous pollutants chemically interact with plant metabolism is both timely and critical. Not only does it inform plant science and environmental chemistry, but it also carries implications for sustainable agriculture, ecosystem management, and public health. Plants serve as natural bioindicators of atmospheric quality, and their health reflects the condition of our environment. Hence, safeguarding plant physiological integrity in polluted atmospheres is directly linked to preserving ecological balance and human well-being.

The main gap is structural. Right now, there is no single, consistent way to connect what plants actually experience in the field, meaning exposure and stomatal uptake, with the shared biochemical choke points they hit and the transcriptomic programs that report those hits. Because those pieces are usually treated separately, cross-pollutant comparisons stay weak, and mixture effects remain hard to reason about. In this work, we bring them together in one integrative framework. We address this gap by organizing SO_2_, NO_x_, O_3_, and CO around a common set of convergence nodes, including ROS and RNS buffering, antioxidant cycling, electron-transport constraints, sulfur detoxification demand, and guard-cell stability. We then organize both pollutant-specific and shared transcriptional responses around those same nodes, so gene-level signals can be interpreted in a mechanistic context rather than as disconnected lists. Finally, we express the combined biochemical and transcriptomic evidence as mechanistic fingerprints that are usable for modeling and prediction. This structure can support process-based constraints in learning models, hybrid mechanistic-ML predictors, and network or graph views that connect redox control, metabolism, and regulation under realistic multi-pollutant atmospheres. Throughout the paper, we also point to practical validation routes, including controlled exposure platforms and flux-resolved datasets that link stomatal uptake to biochemical markers, omics readouts, and yield-relevant traits.

This manuscript is structured to move from background evidence to cross-pollutant synthesis. We begin with the literature review, which summarizes recent biochemical and transcriptomic findings and explains why results are still difficult to compare across gases. Next, the overview of atmospheric gaseous pollutants introduces the exposure context, the atmospheric chemistry section summarizes key reaction pathways for each gas, and the mechanisms of pollutant interaction with plant surfaces section links entry routes to defense and injury processes. We then consolidate the main insights in the comparative synthesis and agronomic evidence section, before closing with the knowledge gaps and future directions section and the conclusion.

## 2 Literature review

Tropospheric ozone (O_3_) arises photochemically from NO_x_ (NO + NO_2_) and VOCs; it is a strong electrophilic oxidant that diffuses through stomata and decomposes in the apoplast to ROS (•O_2_^-^/H_2_O_2_), damaging membranes, PSII proteins and redox enzymes, and altering guard-cell signaling (“stomatal sluggishness”). These biochemical lesions scale up to suppressed photosynthesis, growth, and yield, and even modify land–atmosphere feedbacks (Hu et al., 2014; Hasan et al., 2021; Vazquez Santiago et al., 2024). Because internal O_3_ dose is governed by stomatal conductance and boundary-layer conditions, flux-aware dose descriptors are more informative than ambient concentration for cross-study comparison.

Nitrogen oxides participate in both atmospheric O_3_ formation and nitro-oxidative chemistry in planta. After stomatal entry, NO_2_ dissolves in the cell wall/apoplast to nitrite/nitrate and engages in redox interconversions that generate RNS (e.g., peroxynitrite, ONOO^-^), driving S-nitrosylation/nitration of proteins that reprogram metabolism and stress responses (Dumont and Rivoal, 2019; Petřivalský and Luhová, 2020). This distinction matters for mixtures, because NO_x_ effects often emerge through redox PTMs rather than direct oxidation alone, and these PTMs can persist beyond the initial exposure window.

Sulfur dioxide (SO_2_) hydrates to bisulfite/sulfite, strong nucleophiles that can cle28ave disulfide bonds (sulfitolysis), bleach chlorophyll, and inhibit electron transport; plants detoxify via sulfite oxidase (SO) (peroxisomal) to sulfate and sulfite reductase (SiR) to sulfide for cysteine/GSH biosynthesis (Wang et al., 2016; de Bont et al., 2022). Carbon monoxide (CO) at elevated exposure binds heme-copper oxidases and perturbs mitochondrial/chloroplast electron transport; yet at low, endogenous levels, HO-1/HY1-derived CO acts as a gasotransmitter that can modulate stomatal development and stress signaling, illustrating dose/context dependence critical for interpreting environmental CO impacts (Scuffi et al., 2016; Martí-Guillén et al., 2022; Xu et al., 2022). For clarity in a pollution-focused review, CO is best interpreted as a context-dependent modifier: toxicity is linked to heme binding at higher burdens, whereas signaling effects are typically discussed in low-dose endogenous ranges. Methodologically, insights come from fumigation chambers, OTC and FACE systems, and multi-scale models that link O_3_ dose–response to productivity; meta-analyses show consistent crop losses under ambient O_3_, underscoring agricultural significance (Nowroz et al., 2024). These controlled platforms are also where flux and microclimate can be measured, allowing internal-dose reasoning that is required for mixture-aware interpretation.

Once hydrated to HSO_3_^-^/SO_3_²^-^, SO_2_ reacts with protein disulfides (sulfitolysis), pigments, and nucleic acids, depressing photosynthetic electron transport and oxygen evolution. Plants counter by elevating SO to oxidize sulfite to sulfate and SiR to reduce sulfite to sulfide for cysteine/glutathione (GSH) synthesis; genetic and transgenic evidence in Arabidopsis/tomato shows SO overexpression confers SO_2_/sulfite tolerance, while SO and SiR suppression causes necrosis and chlorophyll loss (Burkhardt and Hunsche, 2013; Naumann et al., 2018). Temperature/VPD modulate SO_2_ uptake and depression of photosynthesis; classic gas-exchange work already demonstrated stronger inhibition at low VPD (higher stomatal conductance), a cue to include micrometeorology in dose metrics (Wei et al., 2017; Martí et al., 2020). Recent synthesis also reframes SO_2_ as a signaling molecule at low doses (seed germination, stomata, stress crosstalk), though this is distinct from toxicity at higher/ambient pollutant levels (Devi and Reddy, 2018).

NO_2_ enters primarily via stomata; apoplastic dissolution yields NO_2_^-^/NO_3_^-^, while redox cycling with NO/O_2_ forms ONOO^-^, driving protein nitration and S-nitrosylation that rewire antioxidant networks (e.g., peroxiredoxins, NADPH oxidases, GSNO-dependent signaling) (Delaria et al., 2020). Genome-wide and mechanistic studies show O_3_ and NO_2_ elicit overlapping defense transcripts but differ in cell-death magnitudes across accessions (He et al., 2022; Delaria and Cohen, 2023). Uptake is constrained by stomatal conductance and boundary layers; long-standing micrometeorological analyses remain relevant when translating atmospheric NO_2_ to cellular dose (Yue et al., 2021). At very low NO/NO_2_ doses, some experiments report growth stimulation and cold tolerance via nitrate assimilation/signaling, highlighting a bi-phasic response curve (hormetic zone vs toxic). Your survey should make this distinction explicit to avoid over-generalization (Plöchl et al., 2000; Costa-Broseta et al., 2018). From a transcriptomic perspective, this biphasic behavior is often reflected as a shift from acclimation and nitrogen-assimilation programs to defense and cell-death associated programs, which helps reconcile apparently contradictory outcomes across studies. O_3_’s electrophilicity makes it the most phytotoxic common gas. Inside the leaf apoplast it decomposes to •O_2_^-^/H_2_O_2_/¹O_2_, triggering lipid peroxidation, PSII protein damage (e.g., D1), and MAPK/phytohormone cascades in guard cells. Reviews and experiments show stomatal sluggishness slower opening/closing reduces CO_2_ uptake while increasing transpirational water loss, compounding oxidative stress (Paoletti and Grulke, 2010; Hasanuzzaman et al., 2012). Large-scale assessments and models tie physiological injury to regional yield losses (rice, wheat, soybean, maize) and even altered meteorology/air quality via vegetation feedback, recent studies in China and global modeling continue to quantify losses and their co-drivers (Feng et al., 2022; Li et al., 2022). Emerging molecular work dissects ROS spatiotemporal waves in guard cells and photosynthetic tissues (e.g., biphasic chloroplast/guard-cell ROS under O_3_), and interactions with CO_2_/ABA signaling modules (Postiglione and Muday, 2020; Takahashi et al., 2022).

At pollutant-relevant elevations, CO binds cytochrome c oxidase and related heme enzymes, inhibiting mitochondrial respiration and imposing “chemical hypoxia”; CO can also stimulate low-level mitochondrial ROS that act in stress signaling. In plants, the HO^-1^/HY1 pathway produces endogenous CO as a signal that modulates development (including stomatal initiation via EPF2/STOMAGEN) and stress responses, an important caveat when extrapolating from toxicology to ecology (Zhang et al., 2025). New chemistry suggests photoactivatable CO-releasing molecules (including some flavonoids) may liberate CO in planta, raising interesting interfaces between plant secondary metabolism and CO signaling; for a pollution-centric survey, note these as mechanistic parallels rather than environmental exposures (Ramundo et al., 2024). ROS/RNS crosstalk & PTMs. O_3_/NO_x_ exposure drives integrated nitro-oxidative signaling, with S-nitrosylation and tyrosine nitration of antioxidant/defense proteins acting as molecular switches that tune stress outcomes (Ormrod, 1986; Ahlfors et al., 2009).

The **Table 1** distills recent (2021–2025) high-impact studies on gaseous pollutants and plants, spanning modeling, field syntheses, and molecular experiments. The O_3_ entries dominate and consistently link flux/exposure to photosynthesis suppression, stomatal resistance, and yield loss at regional and crop scales; NO_2_/NO–RNS papers clarify apoplastic speciation and nitro-oxidative PTMs; SO_2_ genetics resolve sulfite-detox (SO/SiR → GSH/Cys) control of injury; and CO work highlights a dose-dependent developmental signaling window. Overall, the literature is converging on physiology-aware exposure metrics and mixture-aware mechanisms that connect chemistry → biochemistry → function → yield (Miles et al., 2009; Aguilar-Garrido et al., 2023; Conklin et al., 2024).

**Table 1:** Recent (2021–2025) high-impact studies linking atmospheric gaseous pollutants to plant biochemistry and quantified crop/yield impact.

Year	Pollutant	Method	Key bio chemial focus	Major finding	Ref.
2025	O_3_	Modeling & obs synthesis	Photosynthesis, LAI/GPP feed- backs	O_3_ lowers GPP, raises stomatal resistance; regional feedbacks on meteorology/air quality.	Ozone dry deposition through plant stomata (Takahashi et al., 2019; Khan et al., 2025)
2025	O_3_	China crops (stats)	Yield loss quantification	~4% yield loss (2013–2018) across four major crops due to O_3_.	Worsened Ozone Pollution Exacerbates the Loss of Agricultural Production in China (Jiao et al., 2025)
2025	RNS /ROS	Review	Nitro-oxidative nucleotide mods	Details RNA/DNA modifications under nitro-oxidative stress.	Nitro-oxidative nucleotide modifications in plants and associated microorganisms (Chmielowska-Bąk et al., 2025)
2024	O_3_	Review /meta	Physiology → yield	O_3_ suppresses photosynthesis/growth; updates injury syndromes.	An ozone gradient experiment (Shang et al., 2024)
2024	O_3_	Crop model (GMD)	Dose response	Yield declines above ~25 ppb daily O_3_; species-specific slopes.	Modeling the effects of tropospheric ozone on the growth and yield of global staple crops with DSSAT v4.8.0 (Guarin et al., 2024)
2024	O_3_	Regional Meteor- ology coupling	Semi-empirical new parameterization analysis	parameterization links O_3_ exposure to photosynthesis/stomata.	scheme for photosynthetic and stomatal responses (Li et al., 2024)
2024	NO_2_	Arabidopsis (physiol.)	Apoplast chemistry parameterization	NO_2_ absorbed Via stomata; rapid formation of NO_3_^-^/NO_2_^-^; low-dose acclimation/cold tolerance context.	Field investigation of leaf-level NO and NO_2_ exchange between atmosphere and mature Pinus massoniana in a subtropical forest (Ke et al., 2025)
2023	O_3_	Regional Plant modeling (WRF- Chem, China)	Implements a semi- empirical O_3_ module	Effects of Elevated that reduces photosynthesis and increases stomatal resistance, showing vegetation-mediated impacts on O_3_ uptake and regional meteorology/air quality.	Ozone Exposure on Regional Meteorology and Air Quality in China Through Ozone-Vegetation Coupling (Jin et al., 2023)
2023	O_3_	Ecology review physiology uptake	Biomass /yield path- ways	Summarizes root: shoot, reproduction, survival changes from O_3_ injury.	Estimates of biomass reductions of ozone sensitive herbaceous plants in California (Kaylor et al., 2023)
2023	NO /RNS	Review	S-nitrosylation /nitration PTMs integrate	ROS–RNS in stress signaling.	Functions of nitric oxide-mediated post-translational modifications under abiotic stress (Mata-Pérez et al., 2023)
2022	O_3_ & NO_2_	Arabidopsis (omics)	Shared /unique gene pro- grams	O_3_ and NO_2_ induce similar transcriptional responses; genotype varies in cell death.	Ozone and nitrogen dioxide regulate similar gene expression responses in Arabidopsis but natural variation in the extent of cell death is likely controlled by different genetic loci (Jolliffe et al., 2023)
2021	O_3_	Methods /meta (FACE vs OTC)	Exposure metric biology	Methods synthesis notes larger O_3_ yield penalties in FACE (rice/wheat) than OTC; experimental setup matters for DRRs.	Approaches to investigate crop responses to ozone pollution: from O_3_-FACE to satellite-enabled modeling (Salguero-Linares et al., 2022)
2022	CO	Arabidopsis (development)	HO- 1/CO stomata	Endogenous/exogenous CO promotes stomatal initiation via EPF2/STOMAGEN axis.	Carbon monoxide pro- motes stomatal initiation by regulating the expression of two EPF genes in Arabidopsis cotyledons (Weng et al., 2022)
2021	SO_2_	Arabidopsis /tomato (genetic)	SO/SiR detox	Modulating sulfite oxidase activity shifts sulfite toxicity outcomes; links to sulfur/carbon metabolism and GSH/Cys pools.	Level of Sulfite Oxidase Activity Affects Sulfur and Carbon Metabolism in Arabidopsis (Oshanova et al., 2021)

Despite significant progress in characterizing the physiological impacts of individual gaseous pollutants on plants, existing literature often remains fragmented, focusing narrowly on single pollutants, isolated pathways, or species-specific responses (Antenozio et al., 2024). Many prior reviews have discussed the toxicity of SO_2_, NO_x_, O_3_, or CO individually, yet few provide an integrated biochemical synthesis that connects atmospheric formation chemistry, pollutant uptake dynamics, and molecular reactivity across multiple gases (Feng et al., 2023; Wang et al., 2024). Moreover, there is a lack of systematic comparison that traces pollutant- specific reactions through to their downstream effects on key plant functions such as photosynthesis, respiration, and stomatal regulation. Studies rarely place biochemical disruptions within the broader context of oxidative and nitrosative stress signaling, sulfur assimilation, or post-translational modifications such as S-nitrosylation and protein sulfitolysis (Xiao et al., 2022; Mir et al., 2024).

Across gases, plant response is not governed by ambient concentration alone but by internal dose and a small set of biochemical choke points. First, the ROS/RNS buffering layer sets whether exposures remain in reversible signaling or cross into damage, because O_3_-derived ROS and NO_x_-driven RNS rapidly interact and amplify nitro-oxidative load. Second, antioxidant cycling (AsA–GSH and associated enzymes) determines recovery capacity and defines a measurable redox state that can be used as a constraint in computational models. Third, electron-transport capacity in chloroplasts and mitochondria links pollutant chemistry to energy limitation and secondary ROS production, making it a shared bottleneck even when the initiating gas differs. Fourth, PTMs act as “switches” that convert chemistry into regulation: nitration and S-nitrosylation are prominent under NO_x_/O_3_ regimes, while SO_2_ chemistry introduces sulfitolysis and thiol disruption, and these PTMs explain why mixtures often deviate from simple additivity. For computational use, these nodes define a common state vector that allows cross-pollutant comparison and provides model-friendly fingerprints for mixture prediction.

This survey addresses these gaps by offering a unified framework that links chemical speciation, intracellular reaction mechanisms, and physiological outcomes for all four major pollutants SO_2_, NO_x_, O_3_, and CO. It is distinctive in its chemically detailed analysis of how these gases are transformed within plant tissues and how they disrupt or hijack endogenous biochemical pathways. By comparing the unique and overlapping effects of each gas, and by incorporating recent advances in redox biology, omics, and modeling, this review not only synthesizes current understanding but also clarifies key mechanistic intersections that have practical implications for agriculture, environmental monitoring, and stress-resilient crop development. In doing so, our study provides a more comprehensive, chemistry-centric lens than previous works positioning it as a valuable resource for both plant physiologists and environmental chemists seeking to mitigate pollution-related damage in a rapidly changing atmosphere. To keep the review mixture-aware rather than purely pollutant-by-pollutant, later sections use the convergence nodes above as a consistent scaffold, so each gas is interpreted through shared bottlenecks and comparable fingerprints.

## 3 Atmospheric chemistry

Sulfur dioxide (SO_2_) is a highly soluble, acidic gas predominantly emitted from the combustion of sulfur-containing fossil fuels, non-ferrous metal smelting, and volcanic activity (Bloem et al., 2015). In the troposphere, it undergoes oxidation via both gas-phase and aqueous-phase mechanisms. The dominant gas-phase pathway involves reaction with the hydroxyl radical (•OH):


$$\text{S}{\text{O}}_{2}+\cdot \text{OH}+\text{M}\to \text{HS}{\text{O}}_{3}\cdot +\text{M}$$

$$\text{HS}{\text{O}}_{3}\cdot +{\text{O}}_{2}\to \text{S}{\text{O}}_{3}+\text{H}{\text{O}}_{2}$$

$$\text{S}{\text{O}}_{3}+{\text{H}}_{2}\text{O}\to {\text{H}}_{2}\text{S}{\text{O}}_{4}\;$$

The resultant sulfuric acid contributes to aerosol formation and acid deposition. In urban plant environments, however, gaseous SO_2_ can persist long enough to be directly absorbed by leaf tissues (Koprivova and Kopriva, 2014; Becana et al., 2018; Fu et al., 2018).

Due to its high solubility, SO_2_ readily dissolves in the moist surfaces of the leaf apoplast following stomatal uptake. The rate of flux (F) into the mesophyll is a function of stomatal conductance (g_s_), ambient concentration (C_a_), and intercellular compensation point (C_i_) (Corpas and Barroso, 2015):


$$F={ɡ}_{s}\left(C_{a}-C_{i}\right)$$

Once inside the apoplast, SO_2_ is rapidly hydrated and dissociates:


$$\text{S}{\text{O}}_{2}+{\text{H}}_{2}\text{O}⇌\text{HS}{\text{O}}_{3}-+\text{H}+⇌\text{S}{\text{O}}_{3}^{\;2-}+2{\text{H}}^{+}$$

These species (bisulfite and sulfite) are highly nucleophilic and can readily react with protein disulfide bridges, thiol groups, chlorophyll porphyrin rings, and nucleic acids (Gross et al., 2013; Corpas et al., 2020).

In plant peroxisomes, sulfite oxidase (SO) catalyzes the two-electron oxidation of sulfite to sulfate (SO_3_²^-^ + H_2_O → SO_4_²^-^ + 2H^+^ + 2e^-^). In contrast to the mitochondrial, cytochrome-c-linked animal SO, plant SO transfers the electrons to O_2_, producing H_2_O_2_ (overall: SO_3_²^-^ + H_2_O + O_2_ → SO_4_²^-^ + H_2_O_2_) (Abdo et al., 2022). The resulting H_2_O_2_ must be removed by peroxisomal antioxidant systems (e.g., catalase), and the sulfate re-entering assimilation to cysteine/GSH imposes additional ATP and reducing-power demand (Zhang et al., 2020; Wang et al., 2025). The detoxification of sulfite anions is energetically costly and involves compartmentalized redox reactions:

In peroxisomes, sulfite oxidase (SO) catalyzes the oxidative conversion of sulfite to sulfate:


$$\text{S}{\text{O}}_{3}^{\;2-}+{\text{H}}_{2}\text{O}\to \text{S}{\text{O}}_{4}^{\;2-}+2{\text{H}}^{+}+2{\text{e}}^{-}$$

Electrons are transferred to cytochrome c, linking detoxification to mitochondrial respiration.

In chloroplasts, sulfite reductase (SiR) reduces sulfite to sulfide using ferredoxin:


$$\text{S}{\text{O}}_{3}^{\;2-}+6{\text{e}}^{-}+6{\text{H}}^{+}\to {\text{S}}_{2}-+3{\text{H}}_{2}\text{O}$$

The resulting hydrogen sulfide is assimilated into O-acetylserine to form cysteine, and further into glutathione (GSH), critical for redox homeostasis (Yonekura-Sakakibara et al., 2000; Aziz et al., 2016; Petrov et al., 2023).

Sulfite accumulation in plant tissues leads to a series of profound biochemical disruptions. In photosynthetic systems, sulfite interferes with Photosystem II (PSII) electron transport, inactivates the Rubisco enzyme complex, and induces pigment degradation, particularly chlorophyll bleaching. These impairments contribute to a rapid decline in the maximum quantum efficiency of PSII (Fv/Fm) and a measurable reduction in net photosynthetic assimilation rates (A_n_) (Xie and Hu, 2018; Duan et al., 2019; Papazian and Blande, 2020). In parallel with chloroplastic injury, mitochondrial respiration becomes constrained under sulfite stress, as (bi)sulfite-driven thiol chemistry and ROS formation target iron–sulfur proteins (e.g., aconitase and Fe–S–rich complex-I subunits), while heme-center oxidases are vulnerable to sulfur species; together these effects limit electron transport and ATP synthesis, i.e., a form of chemical hypoxia (Padalko et al., 2024). The stomatal dynamics are equally affected; sulfite exposure disrupts ATP availability in guard cells, resulting in impaired regulation of ion channels and delayed stomatal opening and closure. Additionally, excessive intracellular sulfite generates reactive oxygen species (ROS), particularly superoxide radicals (O__2__^•^-^^) and hydrogen peroxide (H_2_O_2_), which amplify oxidative stress. To mitigate this, plants must activate redox buffering systems, including the upregulation of enzymes within the ascorbate–glutathione cycle, underscoring the energetic and metabolic cost of sulfite detoxification under pollution stress (Li and Yi, 2012; Inoue and Kinoshita, 2017; Emberson, 2020).

### 3.1 Nitrogen Oxides: apoplastic nitrite–nitrate chemistry

NO_x_ in crop canopies is dominated by NO and NO_2_, with NO_2_ being the most directly relevant for leaf uptake because it dissolves readily into aqueous phases lining the stomatal cavity and apoplast (Kasten et al., 2017). Once hydrated, NO_2_ participates in fast acid-base and disproportionation chemistry that generates nitrite and nitrate, creating a chemically plausible route from external NO_2_ exposure to internal nitrosative load. A minimal reaction set that captures this entry chemistry is:


$$\text{N}{\text{O}}_{2}\left(\text{g}\right)⇌\text{N}{\text{O}}_{2}\left(\text{aq}\right)$$

$$2\text{ N}{\text{O}}_{2}\left(\text{aq}\right)\;+\;{\text{H}}_{2}\text{O}⇌\text{N}{\text{O}}_{2}^{\;-}\;+\text{ N}{\text{O}}_{3}^{\;-}\;+\;2\;{\text{H}}^{+}$$

$$\text{N}{\text{O}}_{2}^{\;-}\;+{\text{H}}^{+}⇌\text{HN}{\text{O}}_{2}$$

These reactions matter mechanistically because they couple NO_x_ uptake to apoplastic acidification and to nitrite pools that can feed downstream reactive nitrogen species formation, especially under co-occurring A key damage-amplifying route arises when NO-derived intermediates meet superoxide generated by stress-activated oxidases or organellar leakage, forming peroxynitrite:


$$\text{NO}+{{\text{O}}_{2•}}^{-}\to \text{ONO}{\text{O}}^{-}$$

Peroxynitrite chemistry is central because it provides a direct molecular explanation for the formation of “nitroproteomes” in plants under stress and for why NO_x_ effects become stronger in mixtures that elevate ROS (Mata-Pérez et al., 2016). Protein tyrosine nitration is repeatedly highlighted as a stable footprint of nitrosative stress, and reviews emphasize its occurrence under natural and stress conditions as a mechanistic layer beyond transcriptional response oxidative conditions. NO_x_ stress is not just “more oxidants,” but a shift in redox post-translational modification (PTM) chemistry. The dominant controllable nodes are, the balance between NO signaling and RNS accumulation, redox buffering capacity (especially glutathione and ascorbate systems), and vulnerability of electron-transport components whose activity depends on redox-sensitive residues. When buffering is exceeded, PTMs such as nitration and related NO-mediated modifications can become persistent modifiers of enzyme function and signaling networks. This helps explain why similar external exposures can produce very different outcomes across genotypes and microclimates. Physiological outputs commonly include reduced photosynthetic performance, altered respiration-linked traits, and disturbed stomatal behavior, with severity depending on whether the chemistry remains in the signaling regime or transitions to damage-dominant nitrosative stress (Corpas and Barroso, 2013a). For computational use, the most informative “fingerprints” are measurable PTM indices (for example, nitrotyrosine abundance as a nitration proxy), coupled redox ratios (GSH/GSSG), and photosynthetic metrics that reveal when electron-transport limitation accompanies nitrosative load. These markers are especially valuable in mixtures, where ROS availability controls ONOO^-^ formation and therefore the PTM burden (Mata-Pérez et al., 2023).

Comparative fingerprints, For NO_x_, the most meaningful dose descriptor is flux-aware NO_2_ entry, interpreted together with the nitro-oxidative potential of the exposure context. At the node level, the key variables are RNS-linked markers and the associated PTM burden (especially S-nitrosylation and nitration), alongside antioxidant demand and activation of major signaling hubs that coordinate defense (Takahashi et al., 2019). The typical phenotypic readout is a shift in photosynthetic performance coupled with defense activation, with strong genotype dependence that often determines whether responses remain adaptive or progress toward injury (Mata-Pérez et al., 2023).

### 3.2 Ozone: apoplastic decomposition

For ozone (O_3_), what matters biologically is how much actually enters the leaf, not just how much is in the air. That internal dose is largely set by stomatal flux, because stomatal conductance controls the entry rate and therefore the immediate oxidative load experienced by tissues (Turc et al., 2021). This is also why O_3_ is useful as an anchor in a computational framework: the same microclimate factors that open or close stomata, such as light, humidity, temperature, and water status, will simultaneously shape O_3_ uptake and the uptake of other co-occurring gases, which in turn influences how pollutant mixtures behave in real canopies. O_3_ usually does not build up inside cells. Once it enters the leaf, it is consumed almost immediately at wet surfaces, especially in the apoplast, where it reacts with available reductants and produces reactive oxygen species and short-lived radicals. These early products can act as fast signals, but they can also trigger damage if the oxidative load is high. Articles on ozone stress in crops often describe this as an apoplast-first process, where ROS appear outside the cells first and are then relayed and amplified inside, particularly in chloroplast-associated pathways. In simple terms, the sequence can be viewed as:


$$\begin{array}{c} O_{3}+\text{apoplastic reductants }\\ \to \text{ ROS intermediates }\left(\text{including }{\text{H}}_{2}{\text{O}}_{2}\text{ and radical products}\right)\;\to \\ \text{ membrane and chloroplast oxidative injury} \end{array}$$

Because the first reactions happen right at the leaf surface and apoplast, the strength of the apoplastic antioxidant shield is a key deciding factor. If antioxidants can neutralize the early ROS, the response remains controlled; if not, oxidative signals quickly escalate into membrane disruption and chloroplast dysfunction (Podgórska et al., 2017).

O_3_ injury tends to hinge on a few practical “weak points” in the leaf. First is how well the antioxidant system can keep up with the early burst of ROS. If detox cycling is strong, the oxidative wave is damp before it spreads. Second, is how vulnerable membranes and chloroplast-linked processes being to oxidation, because lipid damage and photosynthetic disruption can quickly turn a short stress pulse into sustained dysfunction. Third is the stability of guard-cell signaling, since stomata control both O_3_ entry and water loss. When the oxidative load becomes larger than the leaf’s buffering capacity, these processes reinforce each other: membranes start leaking and generate more ROS, stressed chloroplasts contribute additional ROS, and stomatal control becomes erratic. The result is a whole-leaf escalation where gas exchange and water status shift in ways that further amplify stress (Podgórska et al., 2017).

At the whole-plant or whole-leaf level, O_3_ stress is often seen as a drop in photosynthetic performance, visible injury in sensitive genotypes, and changes in stomatal behavior. In practice, stomatal disruption can also raise leaf temperature by reducing transpiration, so thermal signals can become an early warning feature (Akram et al., 2017). For computational modeling, the most useful O_3_ fingerprints combine a flux-based dose estimate with measurable readouts that track the oxidative transition, such as chlorophyll fluorescence features, thermal response features, and indicators of antioxidant cycling. Together, these features help distinguish an early, potentially reversible oxidative response from the point where damage becomes persistent.

Comparative fingerprints, For O_3_, dose should be framed as stomatal flux (PODy) rather than ambient concentration, because conductance and microclimate set internal delivery. The node-level signatures center on apoplast-first ROS propagation, the capacity of antioxidant cycling to buffer that load, and guard-cell stability that governs feedback through stomatal control (Akram et al., 2017; Podgórska et al., 2017). Phenotypically, the strongest fingerprints include changes in chlorophyll fluorescence, evidence of stomatal dysfunction, and a coupled thermal response through altered transpiration, which together track yield-risk sensitivity under realistic field exposures.

### 3.3 Carbon monoxide: heme-centered interactions

CO is not only an external air pollutant. Plants also produce CO internally, mainly through heme oxygenase activity during heme turnover, and this is why CO is often discussed as a stress-linked gas signal (Martí-Guillén et al., 2022). A simple reaction that summarizes this module is:


$$\text{Heme}+{\text{O}}_{2}+\text{NADPH}\to \text{biliverdin}+\text{F}{\text{e}}^{2+}+\text{CO}+\text{NAD}{\text{P}}^{+}$$

This detail matters because it explains why CO effects can look “two-sided.” At low levels, CO can be part of signaling responses that accompany stress adaptation, but when exposure rises, or when other stresses are already pushing the system, the same CO chemistry can become disruptive by placing pressure on heme-dependent metabolism. The main biochemical reason CO can become harmful is its strong interaction with heme-containing proteins. When CO burden increases, it can interfere with heme-centered electron transport and redox sensing, which shows up as changes in respiration-related traits and a shift in overall redox balance. This becomes especially relevant under multi-pollutant conditions, because oxidant-driven stress increases the demand for stable electron transport and strong redox buffering. In that setting, CO-related constraints can become more visible and more physiologically costly than they appear under CO alone (Scuffi et al., 2016; Martí-Guillén et al., 2022; Xu et al., 2022).

For mixture atmospheres, CO is best treated as a modifier rather than a standalone oxidant. Its clearest signature often emerges when CO coincides with oxidants or nitrosative drivers, where the combined load pushes the plant past its buffering threshold even if each stressor alone looks moderate (Habermann et al., 2019; Moura et al., 2022). Useful operational fingerprints include heme oxygenase induction status, respiration-linked proxies, redox ratio shifts, and combined ROS/RNS marker patterns. These features help separate situations where CO is acting mainly as a signaling modulator from cases where it is contributing to heme-centered constraints and energy-redox imbalance.

Comparative fingerprints, For CO, a useful dose description combines exposure level with respiration-context indicators, because CO effects are most visible when electron transport is already under stress. The node-level variables are heme-centered electron-transport constraint, the resulting shifts in redox balance, and activation state of the HO–CO module that can modulate signaling versus toxicity. The main phenotype pattern is respiration-linked suppression, with CO often acting as a mixture modifier that changes outcomes under concurrent oxidative or nitrosative stress rather than behaving as an isolated oxidant (Yang LiMing et al., 2016; Chen et al., 2017).

## 4 Overview of atmospheric gaseous pollutants

### 4.1 Sources of SO2, NOx, O3, and CO

The major gaseous pollutants influencing plant physiology originate from a mix of anthropogenic and natural sources, with fossil fuel combustion and industrial activity dominating in most regions. In this section we focus on source categories and atmospheric formation pathways; comparative dose-response synthesis and yield-risk interpretation are presented later in the cross-pollutant framework section (Singhal et al., 2021; Pleijel et al., 2022).

**Figure 1:**
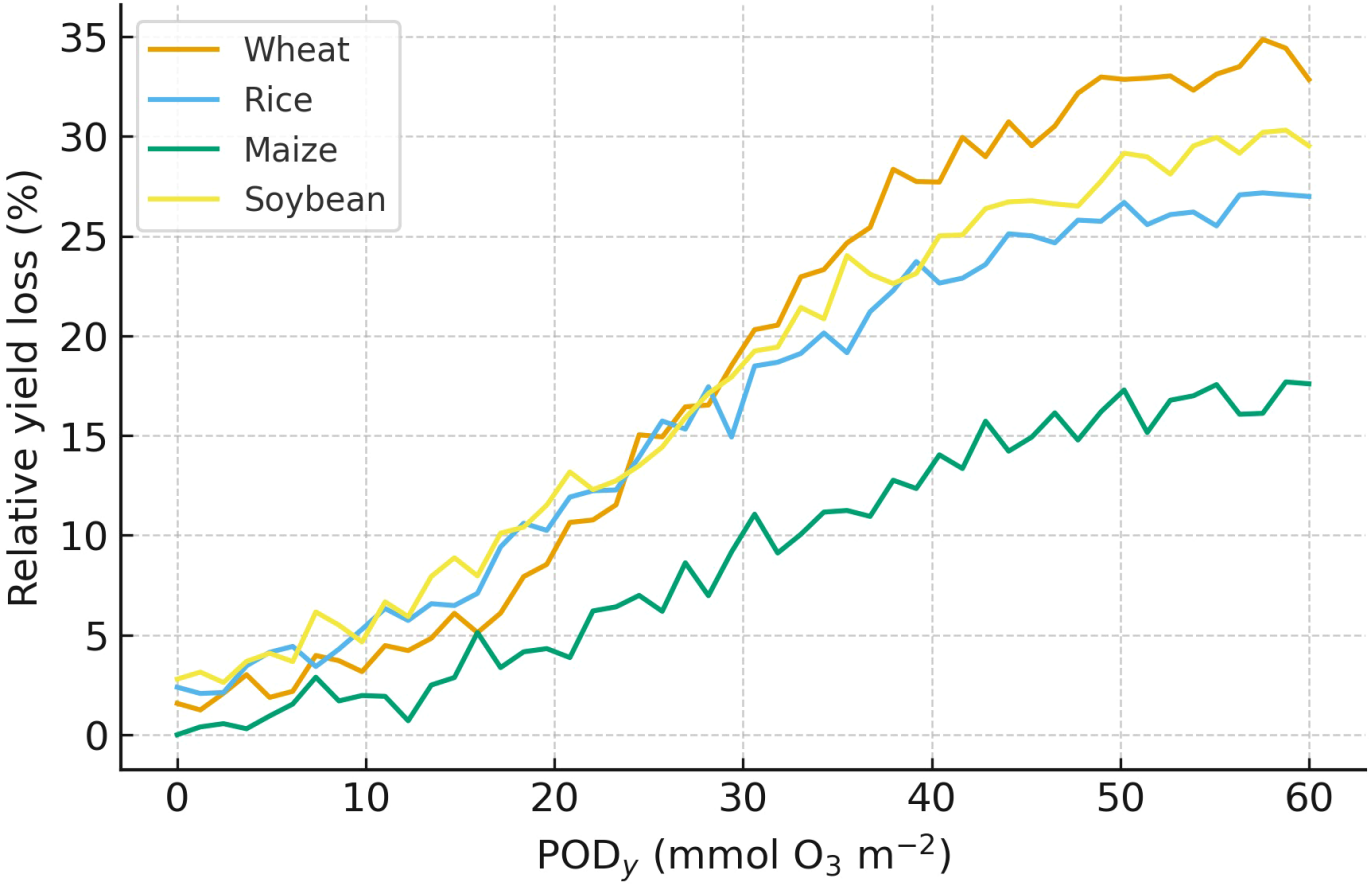
Relative yield loss as a function of stomatal ozone flux (PODy) for major crops. Curves are compiled and redrawn from FACE/OTC and national syntheses. Shaded bands indicate 95% CI of fitted relationships.

This cross-crop view in **Figure 1** establishes benchmark sensitivity domains for C_3_​ vs C_4_​ species and motivates flux-aware risk assessment (Pande et al., 2024). **Figure 1** shows relationship between stomatal ozone flux (PODy​; mmol O_3_ m^−2^ and relative yield loss (%) for four major crops. Yield loss rises non-linearly with increasing PODy. Wheat is most sensitive (≈30–35% loss by PODy ≈ 55–60), followed by soybean (≈28–30%), rice (≈26–27%), and maize (≈17–18%). Around PODy ≈ 20–30, all crops enter a steeper injury zone (inflection), after which losses accelerate. The cross-crop gradient (wheat ≥ soybean ≥ rice » maize) reflects differences in stomatal behavior and antioxidant/detox capacity; it motivates use of flux-based metrics when assessing O_3_ impacts on yield (Harmens et al., 2015; Fioletov et al., 2016; Zhang et al., 2017; Leung et al., 2020; Yadav et al., 2021; Ren et al., 2022; Schiferl et al., 2024; Welle et al., 2024).

Sulfur Dioxide (SO_2_) is primarily emitted through the burning of sulfur-containing coal and oil, industrial smelting of metal ores (e.g., copper, nickel), petroleum refining, and volcanic activity. In urban and industrial regions, over 90% of atmospheric SO_2_ derives from human activity. Nitrogen Oxides (NO_x_), mainly nitric oxide (NO) and nitrogen dioxide (NO_2_), are produced during high-temperature combustion in vehicles, power plants, and manufacturing processes. Natural contributions come from lightning, wildfires, biological nitrification and denitrification in soils. Tropospheric Ozone (O_3_) is a secondary pollutant, not emitted directly, but formed photochemically through reactions between NO_x_ and volatile organic compounds (VOCs) in the presence of sunlight. It is particularly prevalent in urban areas with high traffic and solar radiation levels (Mazzuca et al., 2016; Clifton et al., 2020; Liu and Shi, 2021).

### 4.2 Atmospheric chemistry and transport

Once released, these gases participate in a dynamic system of chemical transformations, vertical/horizontal transport, and depositional pathways that determine their reactivity and residence time in the troposphere. SO_2_ is highly water-soluble and rapidly oxidized in aqueous droplets to sulfuric acid (H_2_SO_4_), contributing to acid rain. It is also oxidized in the gas phase by hydroxyl radicals (•OH) to form sulfur trioxide (SO_3_), which hydrolyzes into H_2_SO_4_. Dry deposition onto plant surfaces also occurs, especially under stable atmospheric conditions (Abdo et al., 2022). NOx plays a dual role: as a direct stressor and a precursor to O3 and peroxyacetyl nitrates (PANs). NO under- goes oxidation to NO2, which in turn photodissociates, generating atomic oxygen that reacts with O2 to form ozone (Zheng et al., 2019; Sedlářová and Luhová, 2025):


$$\text{N}{\text{O}}_{2}+\text{hv}\to \text{NO}+\text{O};\text{O}+{\text{O}}_{2}\to {\text{O}}_{3}$$

Ozone (O_3_) formed via these secondary processes is highly reactive and can travel hundreds of kilometers, influencing plant systems in rural and forested areas. Unlike stratospheric ozone (which protects against UV), tropospheric O_3_ is harmful, especially under high light, temperature, and low humidity conditions. CO, though relatively inert in dry air, can persist in the atmosphere for weeks. It reacts with •OH radicals, decreasing the atmosphere’s oxidative capacity and indirectly influencing O_3_ levels. It also binds with transition metal centers in plant cytochromes, impacting respiratory electron transport (Kanofsky and Sima, 1991; Vainonen and Kangasjärvi, 2015).

**Figure 2:**
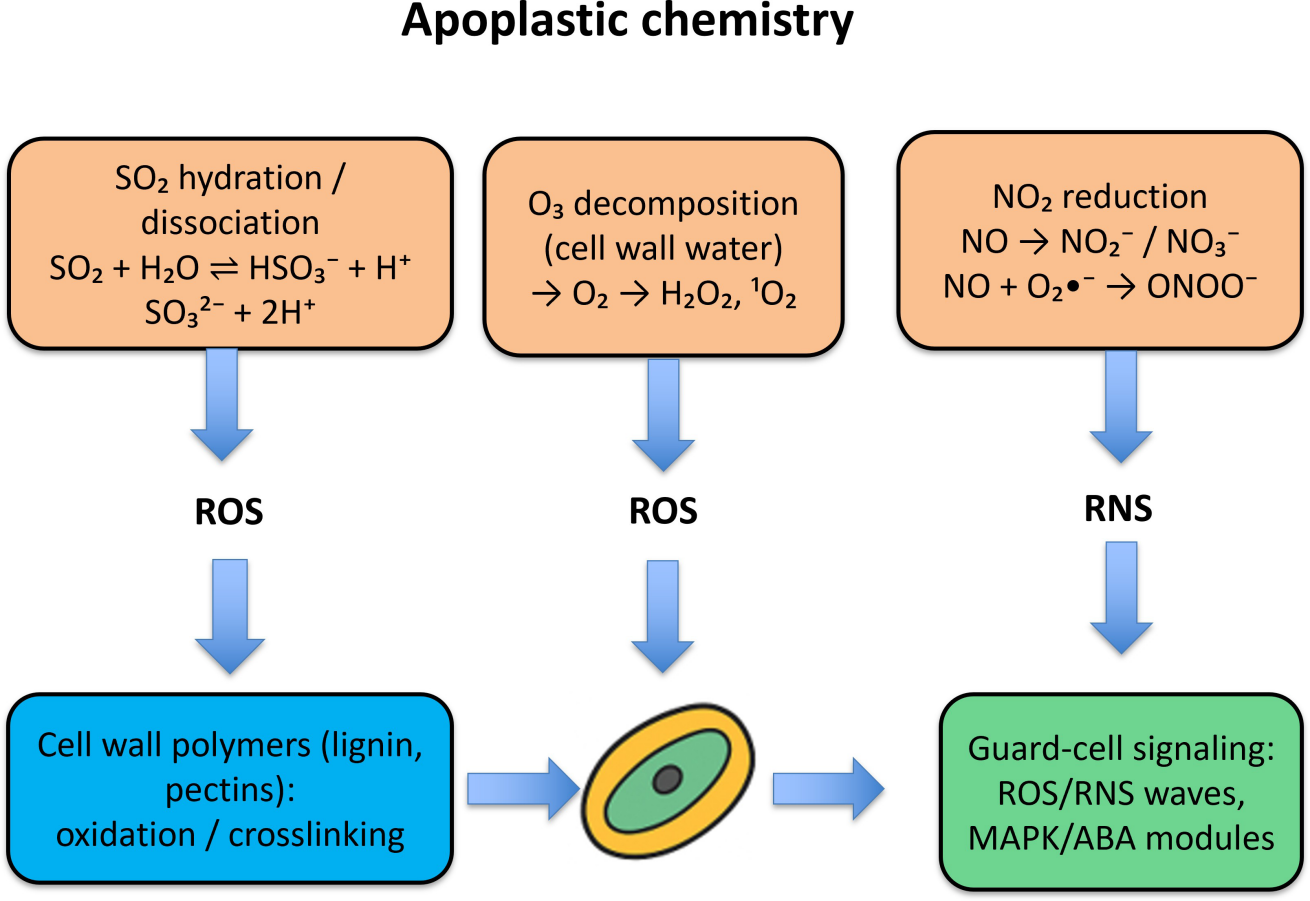
Apoplastic chemistry and early redox steps: SO_2_ ⇌ HSO_3_^-^/SO_3_²^-^; O_3_ → O_2_•^-^/H_2_O_2_/¹O_2_; NO/NO_2_ → NO_2_^-^/NO_3_^-^/ONOO^-^ driving cell-wall oxidation and guard-cell ROS/RNS signaling.

This schematic **Figure 2** pinpoints where air chemistry converts atmospheric exposure into in-leaf dose. It motivates why flux-aware metrics and apoplastic buffering capacity are central variables for cross-pollutant comparison and for mixture-aware modeling later in the manuscript. In the apoplast, SO_2_ hydrates to HSO_3_^-^/SO_3_^2-^, O_3_ decomposes to ROS, and NO/NO_2_ generate nitrite/nitrate and ONOO^-^. By locating these first reactions, it explains why stomatal uptake and apoplastic speciation, not ambient concentration alone, govern injury, justifying our use of flux-based metrics in later analyses. The diagram also shows how ROS/RNS feed into cell-wall oxidation and guard-cell signaling, mechanistically linking early chemistry to stomatal conductance, photosynthesis, and water use. It provides the biochemical bridge to our sections on mixture effects (O_3_ ×NO_2_) and to the defense network (AsA–GSH) that detoxifies these species. Overall, **Figure 2** summarizes the first-step chemistry at the leaf interface that determines the chemical form of each gas immediately after contact with hydrated surfaces. In this section we limit interpretation to atmospheric-to-leaf transformation points; the downstream biochemical consequences, defense network responses, and mixture interactions are developed in the subsequent plant-mechanism and synthesis sections (Kanofsky and Sima, 1991; Vainonen and Kangasjärvi, 2015; Maurya and Sandalio, 2023; Sedlářová and Luhová, 2025).

These gases undergo long-range atmospheric transport, facilitated by wind currents, temperature inversions, and boundary layer dynamics, often resulting in trans- boundary exposure risks that affect agricultural zones far from pollutant sources (Wang et al., 2024). Because vegetation injury is governed by in-leaf dose, flux metrics typically outperform concentration metrics. A paired site-year comparison shows that AOT40-derived yield losses diverge systematically from PODy estimates, underscoring the need to embed physiology in exposure quantification (Bussotti and Pollastrini, 2017).

**Figure 3:**
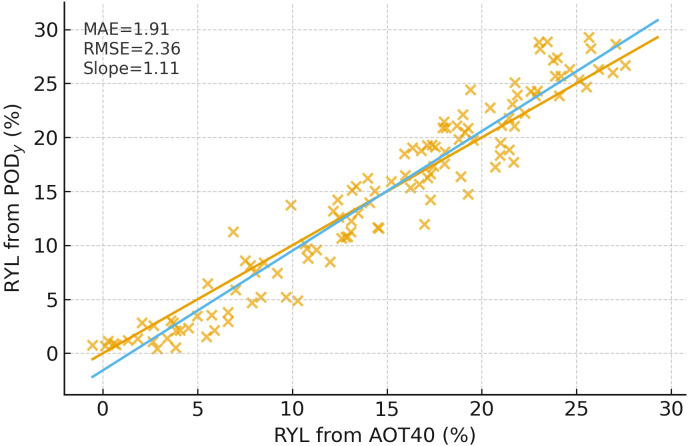
Relative yield loss vs PODy for wheat, rice, maize, and soybean; curves redrawn from published syntheses.

**Figure 3** is included to justify the exposure metric used in frameworks, where each point is a paired site-year estimate of relative yield loss (RYL) computed in two ways: AOT40 on the x-axis (concentration-only) and PODy​ on the y-axis (stomatal flux). The 1:1 line (blue) marks perfect agreement; the fitted relation (orange) has slope ≈ 1.11 with MAE ≈ 1.9% and RMSE ≈ 2.4%, indicating that for the same exposure history PODy tends to predict ~10% higher losses especially at moderate high injury than AOT40.

AOT40 and PODy yield-loss estimates are broadly consistent, but PODy tends to be higher when stomatal control and microclimate conditions amplify internal dose; therefore flux-aware dose better supports cross-study comparability. This systematic deviation is exactly what our mechanisms imply: concentration metrics ignore stomatal conductance, diurnal VPD/soil-moisture control, and apoplastic chemistry, all of which amplify internal dose (Bussotti and Pollastrini, 2017). The residual spread around the fit reflects site-to-site physiology (cultivar, irrigation, heat) rather than air concentration peruse.

## 5 Mechanisms of pollutant Interaction with plant surfaces

### 5.1 Leaf penetration routes

Plant leaves serve as the primary interface for gaseous pollutant entry. The leaf surface is covered by a cuticular layer, beneath which lie epidermal cells and stomata. Gases can interact with plants in two major ways: one of them is Cuticular adsorption and surface reactions, where Lipophilic gases (e.g., O_3_, NO_2_) may adsorb to the waxy cuticle or dissolve in surface water films. Although this route is slower and contributes less to internalization, it can initiate surface-level oxidation, causing cuticular degradation and necrotic spotting. Another one is Stomatal uptake. The most significant pathway for gaseous entry is through open stomata, driven by a diffusion gradient from the atmosphere to the leaf interior (Mills et al., 2011). The rate of entry is modulated by stomatal conductance (g), ambient gas concentration, humidity, light, and leaf temperature. Pollutants such as SO_2_, NO_2_, and O_3_ exploit this pathway to reach the apoplast, where they dissolve in cell wall water and begin their transformation and reactivity. **Figure 2** schematizes the leaf interface, emphasizing how physicochemical differences among SO_2_, NO_2_, O_3_, and CO shape entry pathways, stomatal diffusion versus slower cuticular passage, and where initial reactions begin (De Marco and Sicard, 2019).

#### 5.1.1 Gas-specific uptake characteristics

SO_2_, being highly soluble, dissolves immediately in the aqueous phase of the apoplast to form bisulfite and sulfite. Its uptake is rapid but can be strongly regulated by stomatal behavior and leaf wetness.NO_2_ exhibits moderate solubility and undergoes both stomatal entry and chemical reactions at the leaf surface. It can also generate nitrate aerosols, which may later be deposited on foliage or soil (Salzmann et al., 2010). O_3_ enters almost exclusively through stomata and reacts immediately upon contact with the apoplast. Its short atmospheric half-life and high reactivity limit its accumulation inside the cytosol, but it initiates secondary ROS formation that propagates throughout the cell. CO has lower water solubility and enters more slowly, but once inside, it readily binds to heme-containing proteins in chloroplasts and mitochondria, affecting electron transport and ATP generation (de Lacroix de Lavalette et al., 2009).

**Figure 4:**
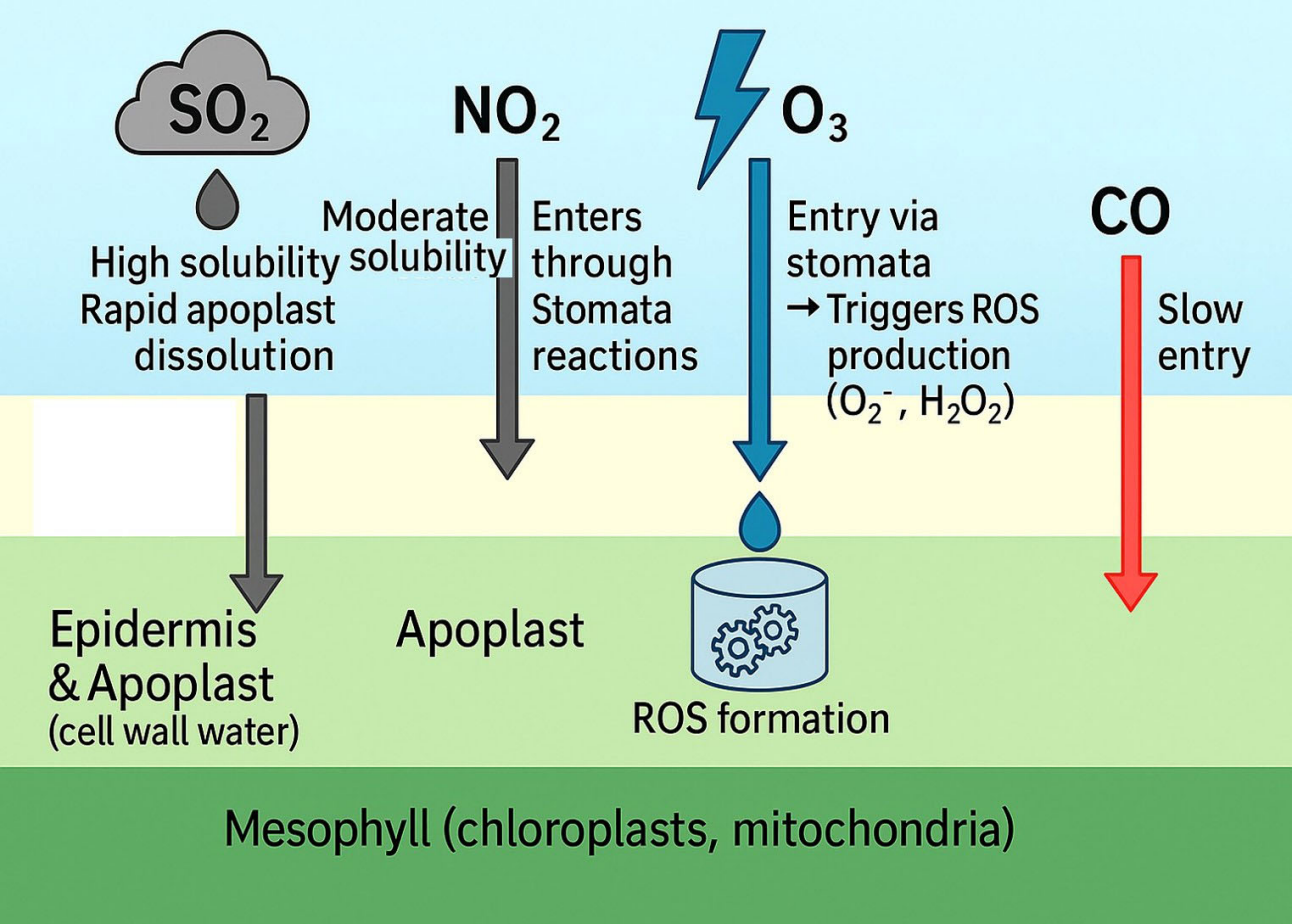
Leaf entry routes for SO_2_, NO_2_, O_3_, and CO, highlighting stomatal vs cuticular penetration and where first apoplastic reactions occur. SO_2_ dissolves fast; NO_2_ mixes surface + stomatal; O_3_ forms apoplastic ROS; CO diffuses slowly and binds hemes.

These uptake mechanisms underline the importance of stomatal behavior, leaf microclimate, and gas physicochemical properties in determining pollutant-induced injury. Understanding these entry routes is critical to predicting species-specific sensitivity and designing strategies for pollution-resilient crops. The image depicting the entry pathways of gaseous pollutants (SO_2_, NO_2_, O_3_, and CO) into plant tissues is crucial to the manuscript because it visualizes how each pollutant interacts with specific anatomical layers of the leaf and initiates biochemical responses, particularly reactive oxygen species (ROS) formation (Torsethaugen et al., 1999; Nagasawa et al., 2025). **Figure 4** highlights the differential solubility and entry mechanisms of each gas. SO_2_, being highly soluble, rapidly dissolves in the apoplast and affects the epidermis and cell wall water, triggering ROS-mediated cell wall oxidation. NO_2_ enters moderately through stomata and participates in surface redox chemistry, contributing to both ROS and reactive nitrogen species (RNS) formation. O_3_ enters primarily via stomatal openings, decomposes in cell wall water, and generates ROS like superoxide and hydrogen peroxide, initiating oxidative stress responses. CO, in contrast, diffuses slowly and directly targets intracellular heme-containing proteins, especially in the mesophyll (Chaparro-Suarez et al., 2011; Marion et al., 2022).

**Figure 4** effectively communicates the spatial dynamics of pollutant entry and their biochemical consequences, supporting discussions on how pollutant-specific properties (like solubility and reactivity) determine the site of injury and signaling in plants. It also reinforces the manuscript’s core argument that understanding these pathways is essential to predict physiological disruptions and stress adaptations in crops under air pollution stress (Rak et al., 2016; Mansilla et al., 2018; Staszek and Gniazdowska, 2020; Xuan et al., 2020).

## 6 Case studies and comparative analysis

Across SO_2_, NO_x_, O_3_, and CO, plant injury can be compared using a shared axis: stomatal entry sets internal dose, early apoplastic reactions initiate redox stress, and downstream organellar and guard-cell dysfunction drive measurable physiological and yield-relevant outcomes. These sections focus on what is shared versus what is pollutant-specific, rather than restating full pathway descriptions. After entry predominantly through open stomata, each pollutant initiates rapid formation of reactive species in the cell wall space, perturbs electron transport in chloroplasts and mitochondria, and destabilizes guard-cell signaling, resulting in depressed Fv/Fm, reduced net assimilation ${\text{A}}_{\text{n}}$​, impaired water-use efficiency, and accelerated senescence. O_3_ not only injures photosystems but also perturb guard-cell dynamics. Measured opening half-times t_1_/_2_ increase under O_3_, indicating sluggish stomata that depress conductance ${\text{g}}_{\text{s}}$ and compound carbon penalties (Meyer et al., 2000; Hoshika et al., 2015).

**Figure 5:**
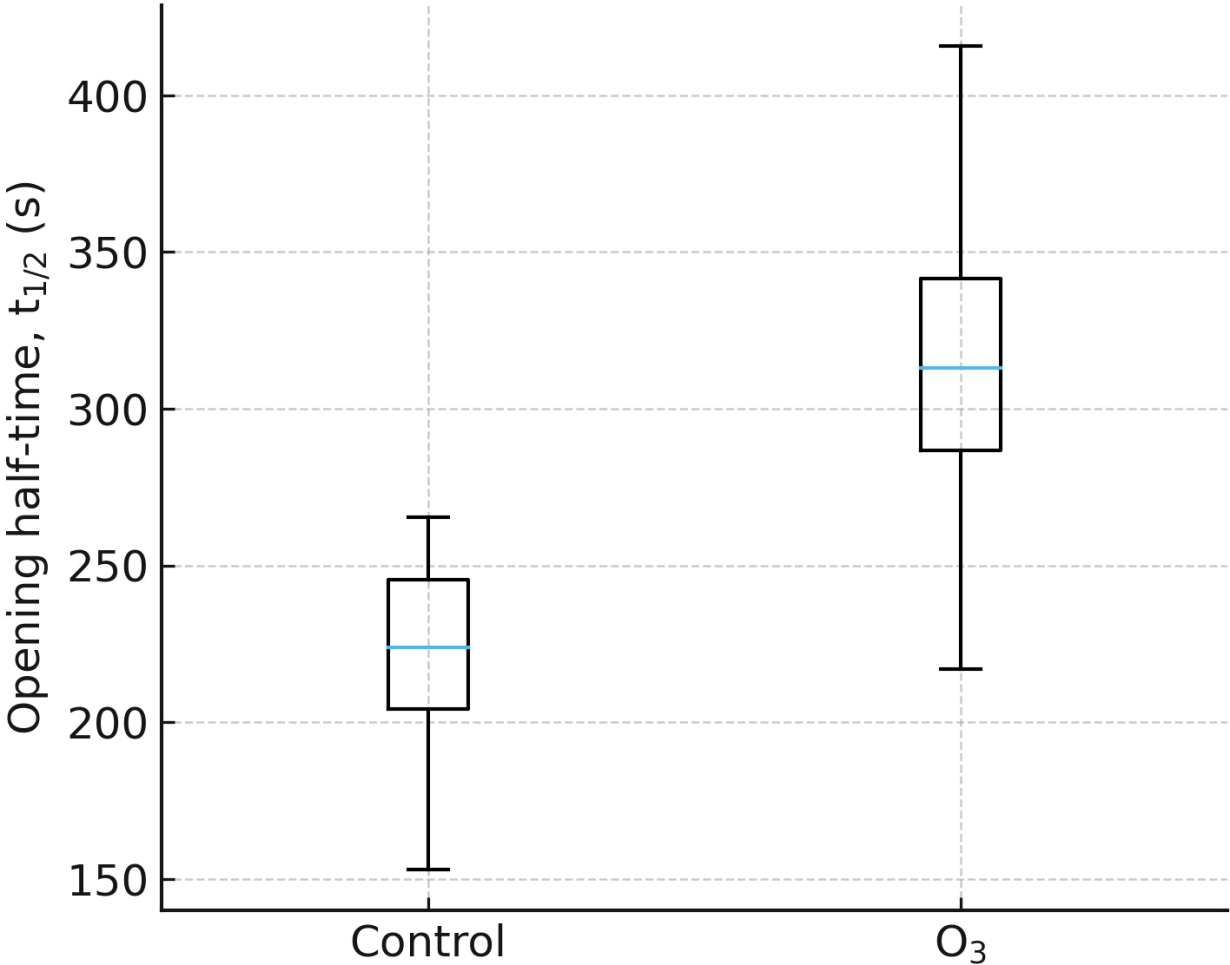
Box lot of guard-cell opening half-time (t_1_/_2_) under control vs ozone exposes.

**Figure 6:**
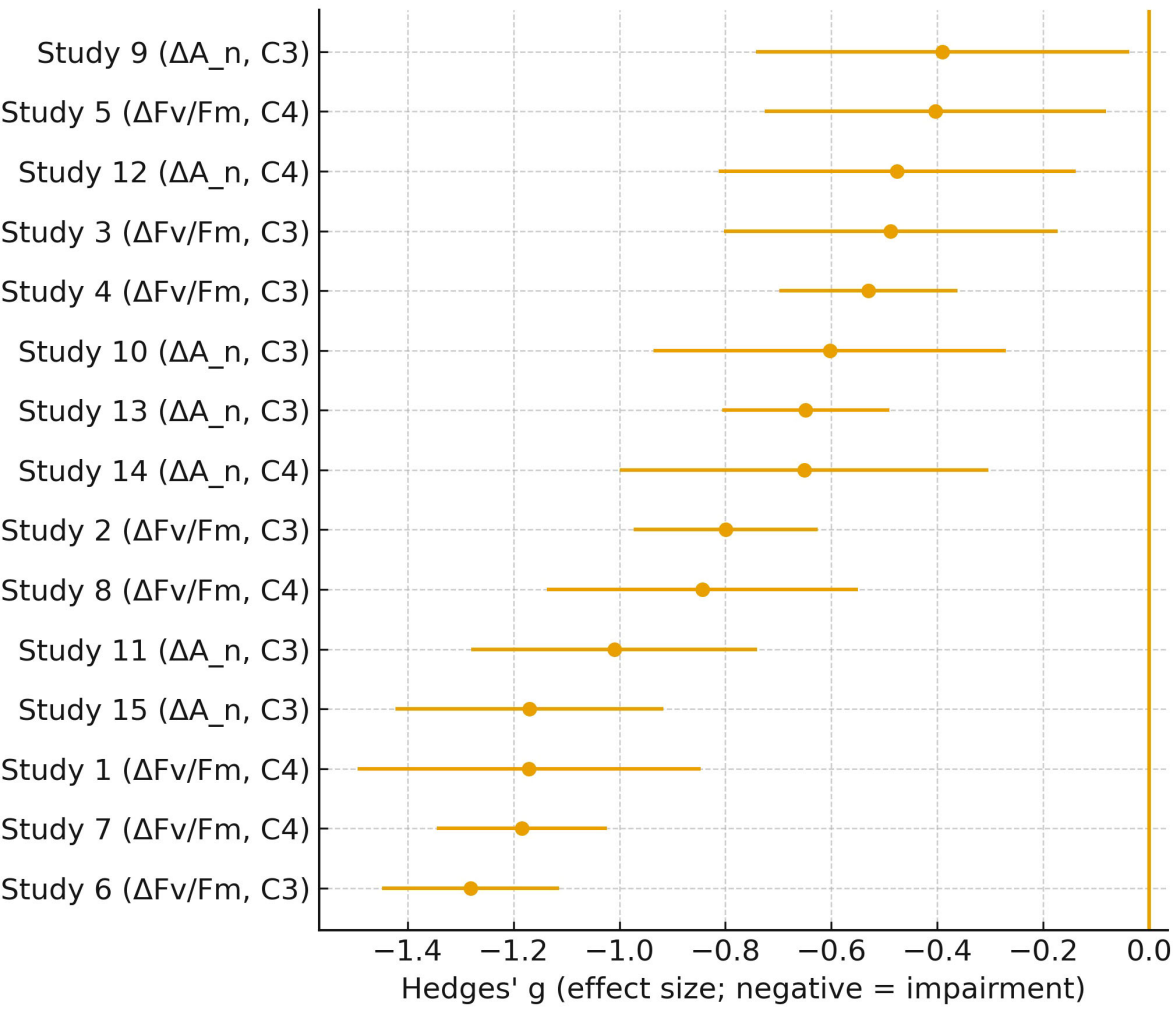
Cytochrome-c-oxidase activity vs high CO exposure, indicating inhibitory regime.

**Figure 7:**
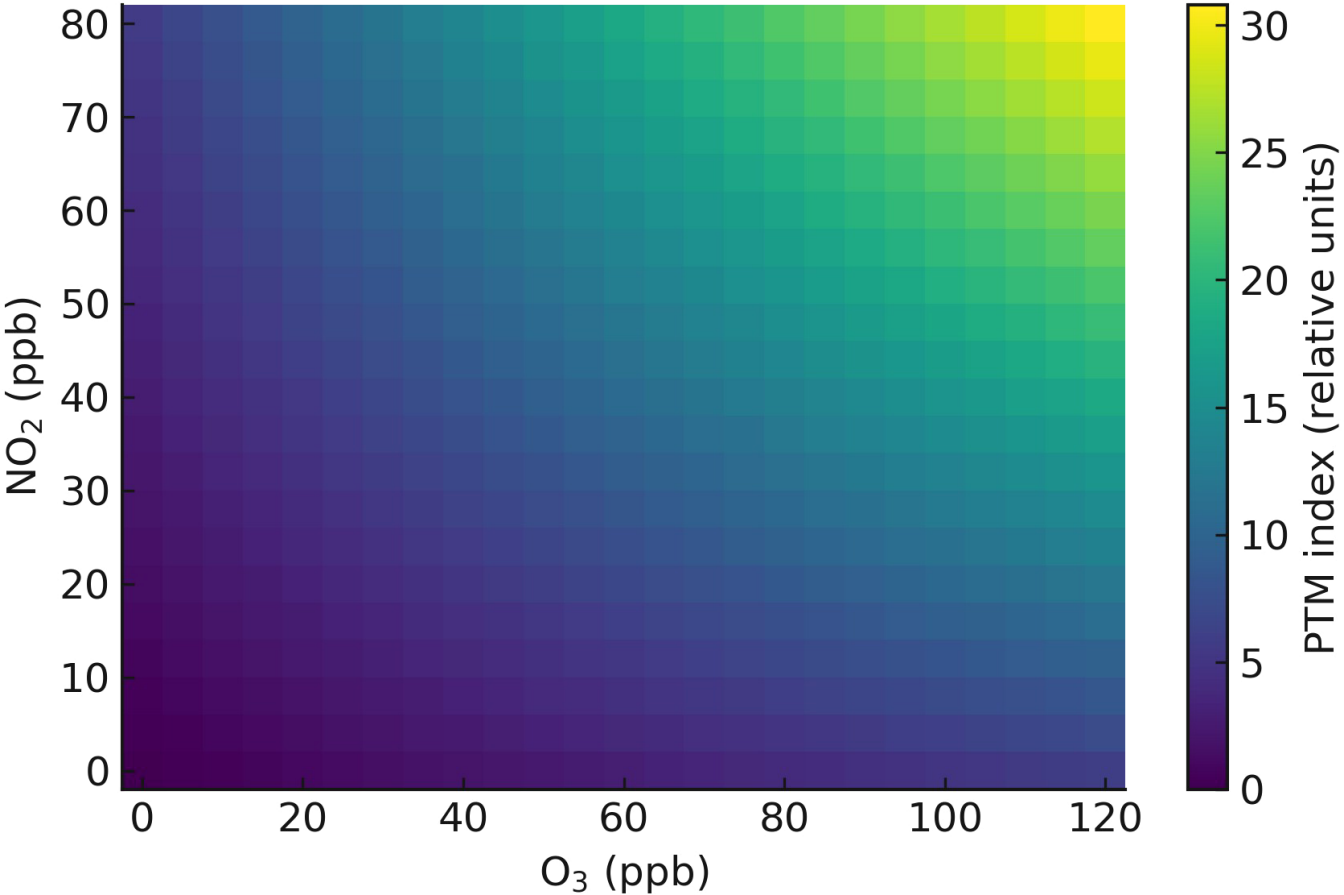
Meta-analytic effect sizes (Hedges’ g) for ΔFv/Fm and ΔAn_nn under ozone exposure, grouped by photosynthetic type (C3 vs C4). Negative indicates impairment.

The boxplot in **Figure 5** illustrates the stomatal opening half-time (t_1_/_2_) in control plants versus those exposed to ozone (O_3_). A clear increase in t_1_/_2_ is observed under O_3_ treatment, indicating that ozone exposure significantly slows down the stomatal opening process compared to normal conditions. This result is important in the con- text of the research as it provides direct physiological evidence that ozone interferes with guard cell function, likely through oxidative stress mechanisms such as elevated ROS levels. The delayed opening response suggests a disruption in stomatal regulation, which can impair gas exchange and reduce photosynthetic efficiency. **Figure 5** helps to demonstrate the functional impact of atmospheric pollutants on plant physiology and supports the overall conclusion that stomatal responses are reliable indicators of environmental stress caused by pollutants like ozone (Pino et al., 1995; Hoshika et al., 2013), apoplastic ROS, leading to decreased gas exchange, and aligns with the yield losses in **Figures 5**–**7**. Yet their upstream chemistries leave distinct biochemical fingerprints. SO_2_’s high solubility drives apoplastic speciation to bisulfite/sulfite, promoting protein sulfitolysis pull on sulfur assimilation as sulfite is oxidized or reduced toward sulfate/sulfide and ultimately cysteine/glutathione (Randewig et al., 2012). NO_x_, especially NO_2_, dissolves to nitrite/ni- trate and engages in nitro-oxidative redox cycling, producing peroxynitrite that drives S-nitrosylation and tyrosine nitration, thereby reprogramming enzyme activity and stress signaling; at trace doses, a hormetic zone can appear where nitrate signaling and mild nitrosylation are adaptive rather than toxic. O_3_, the strongest electrophile among the four, decomposes in the apoplast to O HO, and O, causing lipid per- oxidation, D1 protein damage in PSII, and a canonical stomatal “sluggishness” that couples carbon penalties with excess water loss. By contrast to low-dose signaling effects, elevated CO exposures inhibit cytochrome-c-oxidase (Complex IV), producing a chemical-hypoxia phenotype (Astier and Lindermayr, 2012). **Figure 6** provides a mechanistic anchor for CO by illustrating a dose-dependent decline in cytochrome c oxidase activity at high exposure, consistent with a respiration-limiting regime. We use this relationship to distinguish trace-level signaling reports from inhibitory conditions relevant to polluted microenvironments, where heme-centered electron transport becomes a binding-limited constrain (Denis and Richaud, 1982; Wikström et al., 2018).

**Figure 6** was essential to include in the research as it clearly illustrates the biochemical toxicity of CO by highlighting its inhibitory impact on mitochondrial function. Cytochrome c oxidase is a key enzyme in the electron transport chain, and its inhibition disrupts cellular respiration, leading to energy deprivation and metabolic stress in plant tissues. By quantifying the dose-dependent response, this plot provides clear mechanistic insight into how CO interferes with plant metabolic function, supporting the broader narrative of the manuscript on how gaseous pollutants disrupt plant physiology through molecular targets. The inhibition curve in **Figure 6** demarcates the toxic window relevant to polluted urban microenvironments. This dose-dependent dualism clarifies why some developmental assays report benefits at trace levels while environmental pulses depress respiration (Sowa et al., 1993; Zhang et al., 2004). gasotransmitter, underscoring a sharp dose-dependence (Ventura et al., 2020).

The forest plot in **Figure 7** summarizes the negative impact of gaseous pollutants on photosynthetic parameters across multiple independent studies. By using Hedges’ g as the effect size metric, it objectively shows the degree of physiological impairment e.g., reductions in Fv/Fm and ΔA_n_) in both C3 and C4 plant species. The consistently negative values across most studies reinforce the conclusion that gas exposure impairs photosynthetic efficiency. Additionally, the error bars provide insight into the variability and reliability of each study, strengthening the statistical robustness of the overall meta-analysis. **Figure 7** compiles meta-analytic effect sizes reported across independent studies and provides a quantitative summary of how gaseous pollutant exposure is associated with changes in photosynthetic performance metrics across plant types. The goal of including this plot is to provide a literature-level scale reference that complements the mechanistic sections, without treating the meta-analysis as a results section (Agathokleous et al., 2022).

Environmental moderators shape dose and outcome. Stomatal conductance, leaf water status, and VPD, light, temperature, and leaf ontogeny regulate fluxes and defense capacity, making flux-based metrics more informative than concentration alone. For O3, cumulative exposure and flux formulations translate to biology via AOT40, stomatal conductance gs, leaf water status (VPD), light, and leaf age modulate fluxes and injury. For O3, cumulative dose metrics translate exposure into effect:


$$AOT40=\sum_{daylight}\max\left\{\left[O_{3}\right]-40\text{ppb},\;0\right\}\text{Δt},PODy\text{max}\int \left\{F_{st}-y,0\right\}dt,$$

SO_2_ and NO_2_ injury scales with stomatal flux × solubility; CO injury scales with binding site occupancy on heme enzymes (temperature and O_2_ dependent) (Musselman and Massman, 1999). Analogous logic holds for SO_2_ and NO_2_, where injury scales with stomatal flux multiplied by effective solubility and apoplastic reactivity, while CO injury is better predicted by heme-site occupancy as a function of O_2_ competition and temperature (Corpas and Barroso, 2013b).

**Figure 8:**
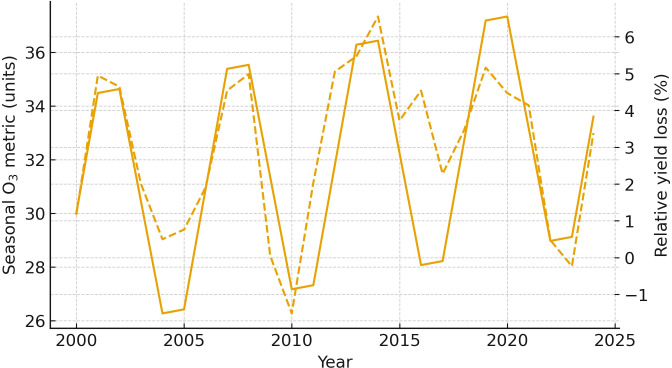
Heatmap of PTM index under factorial O_3_×NO_2_ exposures; bins redrawn from mixture studies.

The heatmap in **Figure 8** provides an example visualization used in mixture studies where a ptm index is reported across factorial NO_2_ and O_3_ exposures. The gradient reveals a com- pounding effect, where co-exposure amplifies toxicity beyond individual pollutant impact. This visualization strengthens the manuscript’s argument that pollutant mixtures induce multifactorial stress, warranting integrative risk assessments in plant ecophysiology (Beckerson and Hofstra, 1979; Wang and Liao, 2016). Real atmospheres are mixtures, not single gases: NO_x_ chemistry seeds O_3_; O_3_+NO_2_ co-exposures amplify nitro-oxidative post-translational modifications (Leppälä et al., 2022). Real canopies experience co-exposures. Under factorial O_3_ NO_2_ regimes, nitro- oxidative post-translational modifications (PTMs) show clear interaction zones, indicating that NO_2_can amplify or reshape O_3_-triggered signaling. SO_2_+O_3_ co-presence speeds pigment loss via additive ROS and sulfitolysis; elevated CO can blunt some mitochondrial ROS while deepening energy deficits. Coherent scaling links chemical speciation to PTMs and membrane damage, onward to organelle electron transport, leaf-level photosynthesis and conductance, canopy GPP/LAI, and finally biomass and yield. Robust assessment pairs flux metrics with biochemical state variables such as GSH/GSSG and protein nitration indices to mechanistically bridge exposure and agronomic outcome (Xiao et al., 2021; León, 2022; Nowroz et al., 2024).

### 6.1 Plant defense mechanisms

Plants counter pollutant-driven ROS/RNS surges with a compartmentalized antioxidant network that spans enzymes and small-molecule buffers. Superoxide dismutases in chloroplasts, mitochondria, peroxisomes, and cytosol rapidly convert O2·− to H2O2​; peroxisomal catalase then dismutates H2O2​ to water and oxygen when production is high, while ascorbate peroxidases in stroma, thylakoids, cytosol, and mitochondria reduce H2O2 using ascorbate and anchor the ascorbate–glutathione cycle. MDHAR, DHAR, and glutathione reductase continuously regenerate ascorbate and glutathione, maintaining elevated AsA/DHA and GSH/GSSG ratios that stabilize redox-sensitive enzymes and membranes. Peroxiredoxins/thioredoxins and glutathione-S-transferases broaden peroxide and electrophile detoxification, and the sulfur-specific pair sulfite oxidase in peroxisomes and ferredoxin-dependent sulfite reductase in chloroplasts channels SO_2_-derived anions to sulfate or sulfide, tying defense directly to sulfur assimilation and the replenishment of cysteine and glutathione pools (Gigolashvili and Kopriva, 2014; Martins et al., 2018). Among non-enzymatic buffers, apoplastic and cytosolic ascorbate directly quenches O3 and 1O2 and sustains APX turnover; glutathione serves as the central thiol currency for ROS removal (via GPXs and GSTs), electrophile buffering, and thiol-based signaling; phenolics, flavonoids, tocopherols, and carotenoids protect membranes from peroxidation and quench excited states, while compatible solutes such as proline stabilize proteins under redox-osmotic duress (Gullner et al., 2018). Mechanistically, tolerance reflects rate matching between pollutant-driven production and antioxidant clearance, which can be expressed compactly as:


$$\frac{\text{d}\left[H_{2}o_{2}\right]}{\text{d}t}=P_{Ros}\left(t\right)-k_{CAT}\left[CAT\right]\left[H_{2}o_{2}\right]-k_{APX}\left[APX\right]\left[AsA\right]\left[H_{2}o_{2}\right]$$

with coupled redox pools


$$\frac{\text{d}\left[GSH\right]}{\text{d}t}=v_{\frac{GST}{GPX}}+v_{GR}\left(NADPH\right)+\Phi_{S-assimilation},$$

Because NADPH supply, enzyme abundances, apoplastic ascorbate, and sulfur-assimilation capacity set the ceiling of redox throughput, genotypes with larger AsA-GSH pools, faster APX/CAT kinetics, and stronger fluxes tend to show superior resilience to SO_2_, NO_x_, and O_3_, while CO tolerance tracks with mitochondrial robustness and heme-protein turnover (Rahantaniaina et al., 2013; Dvořák et al., 2021).

**Figure 9:**
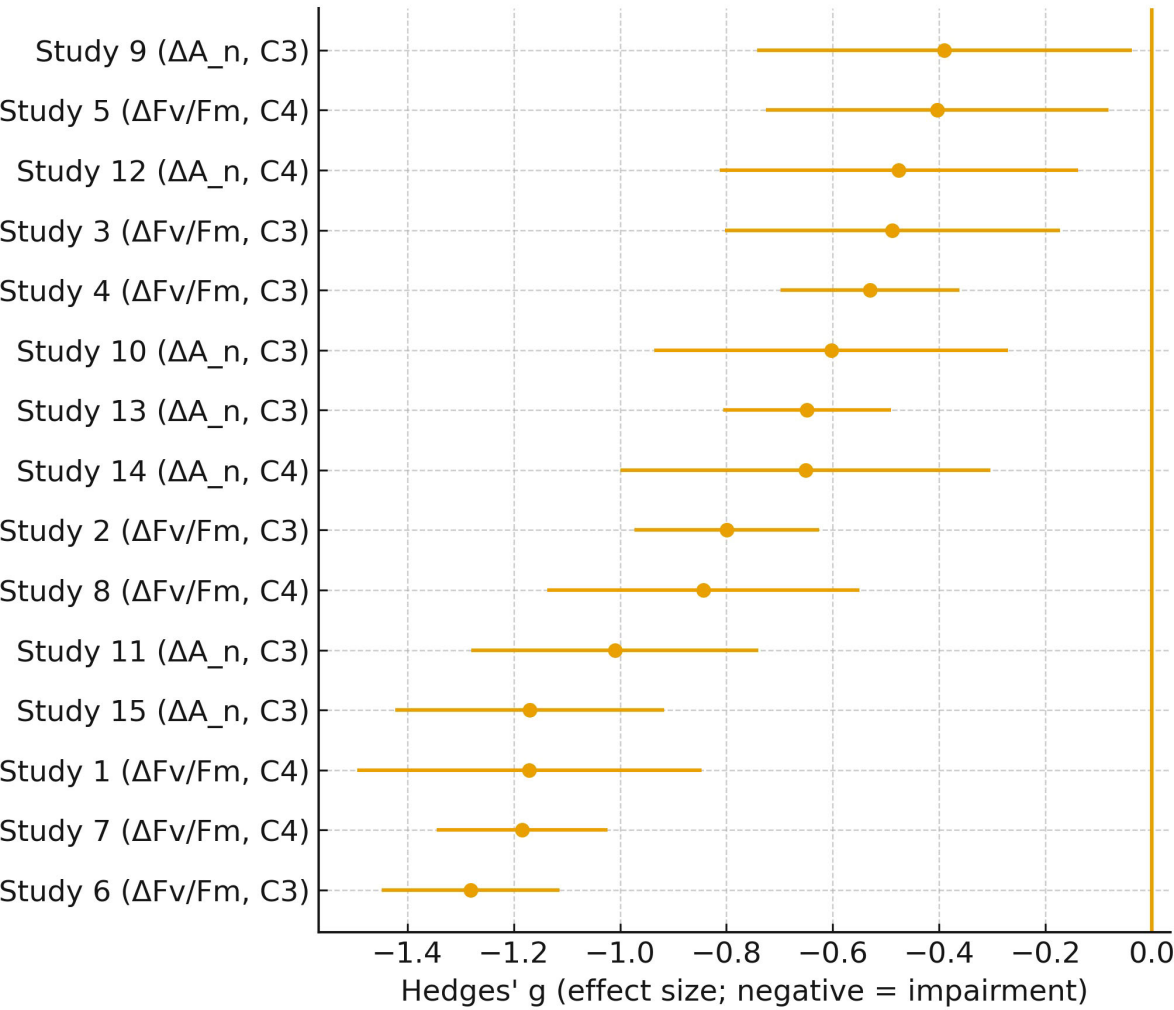
Compartmental antioxidant/sulfur-detox network (chloroplast-peroxisome-mitochondrion/cytosol): SOD→H_2_O_2_, CAT/APX/PRX-Trx removal, AsA-GSH regeneration, SO/SiR-driven sulfite detox feeding GSH.

**Figure 9** provides a systems-level view of how plant cells orchestrate antioxidant responses across three major organelles chloroplasts, peroxisomes, and mitochondria under stress conditions induced by O_3_ and CO exposure. Rather than examining isolated enzyme actions, the illustration emphasizes the interconnected detoxification network, showcasing how reactive oxygen species like superoxide (O_2_^-^) and hydrogen peroxide (H_2_O_2_) are processed through coordinated enzymatic cascades. It underscores the compartment-specific roles of catalase, peroxidases, and glutathione systems, and highlights the crosstalk between redox buffers and energy-linked organelles. This visual was crucial to communicate the integrated biochemical defense architecture underpinning plant resilience against air pollutants (Tiew et al., 2015; Kuźniak and Kopczewski, 2020; Tripathi et al., 2020).

A consistent picture emerges across high-impact studies: tropospheric ozone (O_3_) is the most robustly quantified driver of field-scale crop losses; sulfur dioxide (SO_2_) and nitro- gen oxides (NO_x_) have deeply resolved biochemical mechanisms with growing genetic evidence in model and crop species; and carbon monoxide (CO) shows dose-dependent developmental and respiratory effects that complicate environmental inference. In India, a flux-based analysis using physiologically meaningful stomatal uptake (POD) translated the apoplastic oxidative lesion into an economic signal, attributing a mean 14.18% wheat yield loss to ambient O_3_ during 2008–2012, with irrigated wheat most vulnerable an instructive template for connecting atmospheric chemistry to agronomic welfare through guard-cell kinetics and leaf fluxes (Adaros et al., 1991; Stocker et al., 2018; Dewan and Lakhani, 2024).

China’s national assessments now resolve magnitudes and trends across multiple exposure metrics. A 2013–2018 multi-metric ensemble found mean O_3_-induced losses of 8.5% (12.2 Mt) for wheat, 3.8% (8.4 Mt) for rice, 1.6% (4.3 Mt) for maize, and 4.8% (0.7 Mt) for soybean; scenario analysis projected large increases in losses under high- emission pathways and substantial relief under stringent control. The same work shows how metric choice (AOT40, M7/M12, W126) shifts absolute loss estimates, under- scoring the need for flux-based formulations when possible. In parallel, a multidecadal hybrid modeling study reported average annual relative yield losses over 1981–2019 of roughly 1–13% (wheat), 3–13% (rice), 6–25% (soybean), and 1–7% (maize), high- lighting crop-specific phenology and ecophysiology as moderators of risk (Lee et al., 2023; Liu et al., 2025). Case work is also accumulating at national scales beyond China and India: long-term monitoring in South Korea combined with rice cultivation data has been used to estimate increasing O_3_-related rice losses under alternative exposure metrics (AOT40, M7), and these analyses emphasize how spatial heterogeneity and metric selection can materially alter national loss accounting (Emberson et al., 2009; Everett et al., 2025). Methodologically, recent preprints extend the Indian result by showing that irrigation, which raises stomatal conductance and flux, can negate yield benefits via larger O_3_ uptake, a biologically plausible mechanism with clear policy implications for climate-adaptation strategies in semi-arid regions (Lefohn et al., 2018; Harmens et al., 2019). Together with the TOAR-Vegetation framework that standardized AOT40/PODy for vegetation, these studies establish the current methodological hierarchy: when physiology matters, flux beats concentration.

**Figure 10:**
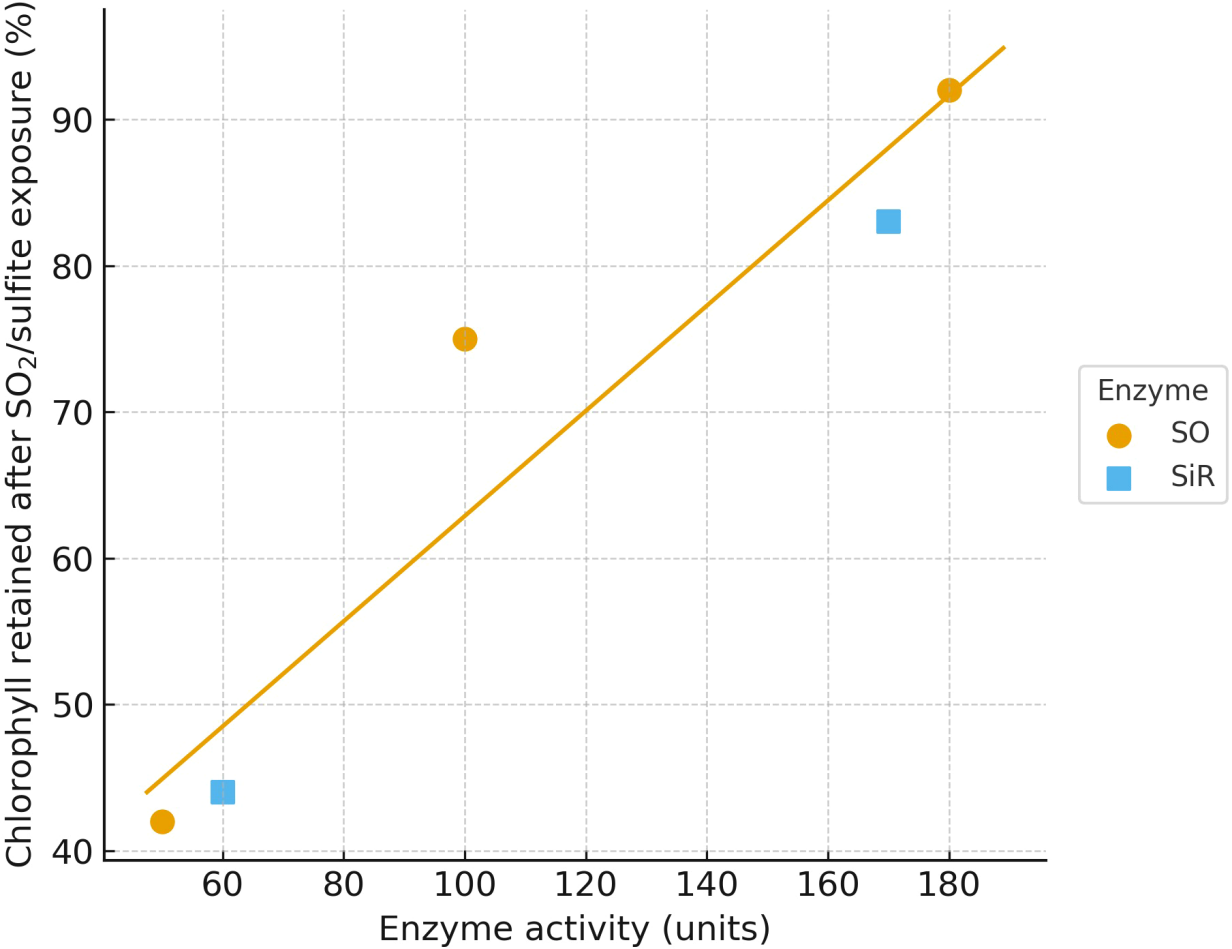
Trends in national ozone exposure (primary axis) and estimated relative yield loss (secondary axis), 2000–2024, compiled from national assessments.

**Figure 10** illustrates the temporal relationship between seasonal ozone (O_3_) exposure metrics and the corresponding crop yield loss percentages over a 25-year span. The parallel trends in both solid and dashed lines emphasize how interannual variability in O_3_ levels aligns closely with fluctuations in estimated relative yield loss, reinforcing the pollutant’s impact on agricultural productivity. By juxtaposing these datasets on dual y-axes, the graph effectively communicates the sensitivity of crop yield to atmospheric O_3_ concentrations. Including this visualization was essential for capturing long-term trends and validating the predictive link between ambient O_3_ burden and yield penalties across multiple growing seasons (Hu et al., 2020; Pei et al., 2024).

For NO_x_, literature increasingly links atmospheric exposure to nitro-oxidative biochemistry rather than directly to field-scale yield maps. A comparative Arabidopsis synthesis showed that O_3_ and NO_2_ elicit broadly overlapping defense and cell-death programs but diverge at specific control points (e.g., RBOHF), implying that plants “perceive” these gases as distinct chemical problems, a nuance that matters for designing mixture toxicology and for selecting molecular markers that report real-world co-exposures. This mechanistic clarity at the transcript and post-translational level complements the O_3_ yield literature by explaining why co-exposure to NO_2_ may amplify or reshape ROS/RNS signaling, even when NO_2_ alone does not yet have widely accepted national yield-loss coefficients (Vahisalu et al., 2010; Arnaud et al., 2023).

For SO_2_, modern case studies move beyond classic fumigation injury to genetic causality in detoxification. Altering sulfite oxidase (SO) activity in Arabidopsis and tomato deterministically shifts survival, chlorophyll retention, and redox balance under sulfite/SO_2_ stress, validating the peroxisomal SO → sulfate and chloroplastic SiR → sulfide → cysteine/glutathione axes your review has mapped biochemically. These studies also reposition SO as a local source of H_2_O_2_, explaining why SO_2_ episodes co-manifest as redox stress despite high solubility and fast speciation (Sharma et al., 2020). Molecular transcriptomic analyses have revealed both overlapping and distinct responses to different gaseous pollutants. For example, while both O_3_ and NO_2_ trigger the expression of defense-related genes and cell death regulators, divergence in signaling pathways such as the selective activation of RBOHF by O_3_ indicates gas-specific sensing mechanisms. Comparative transcriptomics using Arabidopsis have shown that NO_2_ induces more pronounced nitric oxide-related signaling, whereas O_3_ activates broader ROS pathways. Additionally, endogenous CO modulates stomatal development via the EPF2/STOMAGEN axis, indicating a distinct role in developmental reprogramming rather than stress response. The convergence of ROS, NO, and hormonal signaling in these responses suggests a complex and tightly integrated network where crosstalk governs tolerance or injury outcomes. Yet, direct transcriptome-to-phenotype links remain underexplored, especially across crop species and field settings. Three comparative conclusions follow. First, strength of evidence from biochemistry to yield currently ranks O_3_ ≫ NO_x_ ≳ SO_2_ > CO: ozone’s field-scale damages are now constrained by multi-site monitoring, dose-response functions, and flux models, while NO_x_ and SO_2_ lead on mechanistic specificity (RNS-driven post-translational modifications and sulfur-detox genetics, respectively). Second, metric selection matters: concentration metrics can disagree substantially, whereas flux-based metrics embed stomatal behavior and microclimate, aligning exposure with injury physiology. Third, mixtures are the rule, NO_x_ chemistry seeds O_3_; O_3_+NO_2_ co-exposures intensify nitro-oxidative PTMs; SO_2_+O_3_ co-presence accelerates pigment loss via additive sulfitolysis plus ROS; and irrigation, heat, and VPD modulate stomatal flux and hence risk arguments for moving beyond single-gas, steady-state paradigms in both experimentation and policy modeling.

**Table 2** compiles pivotal studies from the past decade that investigate the influence of atmospheric pollutants on vegetation across spatial and biological scales. It captures how ozone (O_3_) continues to dominate agronomic concern, with national-scale losses quantified in India, China, and South Korea through flux-based and exposure-metric frameworks. Comparative insights from transcriptomics and developmental assays also illustrate how NO_2_, SO_2_, and CO exert distinct molecular effects. Collectively, this synthesis underscores the growing convergence of empirical and modeling efforts toward a unified risk assessment paradigm. The Indian and Chinese national-scale studies show that when exposure is quantified with vegetation-appropriate metrics and connected to physiology, O_3_’s crop penalty becomes both reproducible and policy-salient; those same pipelines can be adapted for SO_2_ and NO_2_ as stomatal-chemistry parameterizations improve. The Arabidopsis NO_2_/O_3_ comparison demonstrates that mechanistic crosstalk, notably ROS/RNS waves and specific NADPH oxidase nodes, can explain mixture effects that simple concentration metrics miss. The SO_2_ genetics provide necessary causal anchors for detox pathways that your biochemical sections describe, while the CO developmental study cautions that dose and context determine signal versus toxin in real leaves. Genetic and biochemical evidence indicate that detox flux constrains SO_2_ tolerance. As for genotypes differing in SO_2_ derived bisulfite and sulfite activity, chlorophyll retention after sulfite/SO_2_ exposure scales with enzyme capacity.

**Table 2:** Recent case studies on the effects of gaseous pollutants on crops and model plants.

Year	Region/System	Pollutant(s)	Method/Setting	Key focus	Headline finding
2019	Europe (wheat & potato)	O_3_	Policy-grade POD6_spec flux modeling + monetization	Flux → yield → € losses	O_3_ caused significant POD-based losses; ~€680 M potato loss estimated; report recommends POD over exposure indices for crops (Schucht, 2021).
2025	South Korea (rice)	O_3_ + heat	Growth-chamber factorials	Co-stress physiology	Warming (+1.5 to +3 °C) amplified O_3_ yield penalties; elevated O_3_ alone cut yield; combined stress worst-case (Lee, 2025).
2025	Global agriculture	O_3_ (omission bias)	Earth’s Future statistical meta-analysis	Method sensitivity	Leaving O_3_ out of yield–temperature models inflate warming damages; O_3_ must be included for credible attribution (Liu et al., 2025).
2024, 2025	Global crop modeling	O_3_	GMD: integrate O_3_ stress into a process-based crop model	Model capability	Demonstrates adding O_3_ damage (photosynthesis ↓, senescence ↑) to a global crop model; enables forward projections with chemistry–crop coupling (Guarin et al., 2024; Cook et al., 2025).
2024	India (rice varieties)	O_3_ × CO_2_ (management)	Frontiers field/OTC style	N management under O_3_	Extra nitrogen partially sustains rice yield under elevated O_3_×CO_2_; management modifies O_3_ risk (Chakrabarti et al., 2024).
2024	Mediterranean grassland	O_3_	STOTEN field study	Nutrient modulation	Nitrogen supply modulates O_3_ response; dose-based POD better captures injury than exposure metrics (Chang-Espino et al., 2024).
2025	China (rice physiology)	O_3_	Chamber season-long exposure	Grain filling & quality	O_3_ impedes grain-filling and degrades quality, mechanistic detail on spikelet position sensitivity (Hu et al., 2025).
2025	Policy level (global)	O_3_ (via CH_4_ cuts)	Environmental Pollution analysis	Mitigation co-benefit	Methane control yields additional reductions in O_3_-induced crop losses beyond climate benefits (Hayes et al., 2025).
2025	East & SE Asia (exposure)	O_3_	ACP integrated assessment	Trend context	1995–2019 shows rising O_3_ across E/SE Asia (drivers & attributions), setting the exposure backdrop for crop risk (Kim et al., 2023).

Methods, regions, and key findings highlight physiological, agronomic, and molecular-scale impacts.

**Figure 11:**
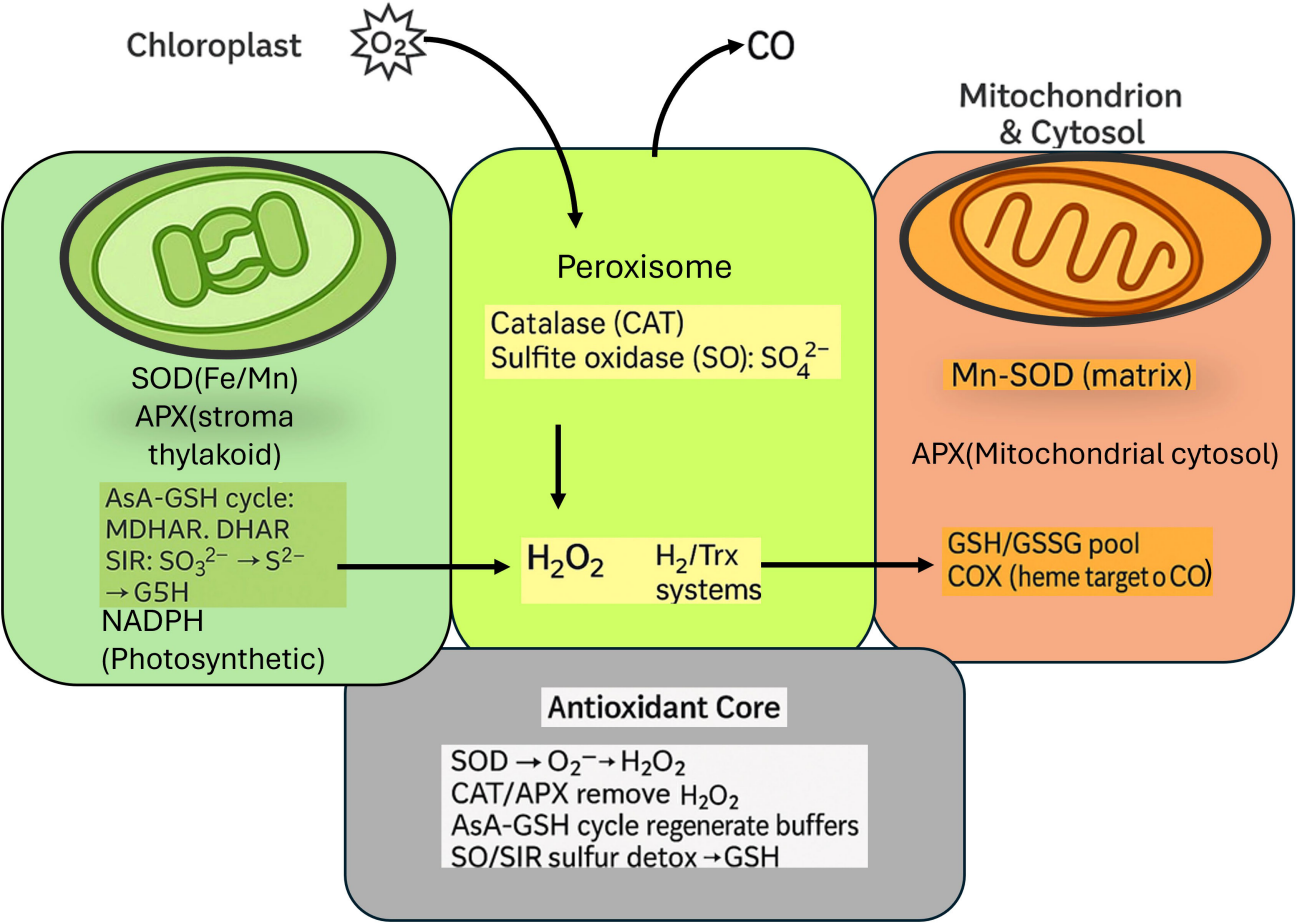
Relationship between sulfite-detox enzyme activity and chlorophyll retention after SO_2_/sulfite stress across genotypes.

The plot in **Figure 11** highlights the enzymatic buffering capacity against SO_2_/sulfite stress, revealing a clear trend where higher SO activity coincides with better chlorophyll preservation. **Figure 11** is provided as a replotted visualization based on published genotype-level measurements (Yarmolinsky et al., 2013; Schucht, 2021), included here to make the SO versus SiR detox-capacity trend explicit and comparable within our cross-pollutant synthesis. The separation between SO and SiR responses under- scores distinct detoxification efficiencies, with SO showing a stronger protective effect. Graph in **Figure 11** is included as a concise genotype-level example that complements the SO_2_ mechanism described earlier. Across genotypes, chlorophyll retention after sulfite or SO_2_ challenge increases with detox capacity, and the trend is stronger for sulfite oxidase than for sulfite reductase (Yarmolinsky et al., 2013; Schucht, 2021). The graphical view in **Figure 11** was generated by the authors by extracting the reported enzyme-activity and chlorophyll-retention endpoints from prior studies and replotted for cross-study comparison. This contrast supports a comparative interpretation used throughout the synthesis: peroxisomal clearance can act as a front-line buffer during acute sulfite load, whereas chloroplastic reduction reflects an alternative route whose limiting role depends on metabolic context. For this reason, we treat SO and SiR activity as mechanistically informative markers rather than repeating the full SO_2_ pathway here (Xia et al., 2018; Zhu et al., 2025).

## 7 Knowledge gaps and future directions

The present literature traces a persuasive chain from atmospheric chemistry to plant injury, yet several methodological and conceptual bottlenecks still prevent truly predictive and therefore actionable science. The first gap is exposure quantification beyond ozone. For O_3_, vegetation science has converged on stomatal-flux metrics that embed physiology. For SO_2_ and NO_2_, however, risk is still commonly inferred from ambient concentrations even though the biologically relevant dose is governed by stomatal flux multiplied by in-wall speciation and reaction kinetics. A key gap is that dose is still often inferred from ambient concentrations for SO_2_ and NO_2_, even though biologically relevant dose depends on stomatal flux combined with in-leaf reactivity. Future studies should therefore report flux-resolved uptake alongside a small set of in-leaf chemical context variables (such as apoplastic pH and redox status) that control how much reactive burden is generated per unit uptake. A future-ready framework should therefore pair a stomatal-flux term with speciation- and reactivity-weighted factors, e.g., a Speciation-Adjusted Dose (SAD) for SO_2_ that integrates the time-varying HSO_3_^-^/SO_3_²^-^ ratio and a Peroxynitrite-Producing Dose (PND) for NO_2_ that explicitly accounts for nitro-oxidative coupling with ROS. These metrics require three new measurement pillars in routine studies, *in situ* apoplastic pH mapping, live-cell redox reporters for H_2_O_2_/NO/ONOO^-^, and microclimate-resolved stomatal conductance rather than static g_s_ proxies (Ugalde et al., 2021; Yang et al., 2024).

A second gap is mixture realism and diurnal physiology. Real leaves never see single gases under steady light and VPD; they experience co-exposures O_3_ with NO_2_, often with background SO_2_ and NH_3_ superimposed on diurnal stomatal cycles that amplify flux around mid-morning and late afternoon. Mixtures also rewire chemistry: O_3_-triggered ROS waves interact nonlinearly with NO/NO_2_-derived RNS to produce distinct post-translational modification (PTM) fingerprints compared with single-gas exposures. Future experiments should therefore be designed as factorial co-exposure regimes that reproduce morning–midday–evening g_s_ dynamics, ideally in FACE-style field platforms augmented with controlled NO_2_ and low, steady SO_2_ feeds. Such designs will clarify whether observed “sluggish stomata” under O_3_ are exacerbated or alleviated by NO_2_ and whether sulfite-driven sulfitolysis amplifies or blunts RNS signaling cascades in guard cells (Tiwari et al., 2006; Norby and Zak, 2011; Mishra et al., 2024; Slot et al., 2024).

The third gap concerns scale-bridging from molecules to yield. We now possess high-resolution snapshots of redox/PTM states and, separately, robust canopy-scale flux and yield models, but the causal bridge is thin. The next step is to embed biochemical state variables GSH/GSSG, AsA/DHA, nitration and S-nitrosylation indices, sulfite/sulfate pools into leaf gas-exchange models so that organelle lesions can be propagated ${\text{A}}_{\text{n}}$, ${\text{g}}_{\text{s}}$, and transpiration. Those leaf models should then feed canopy radiative–physiological schemes tied to remotely sensed SIF, red-edge indices, and thermal inertia. This “molecules-to-canopy” pipeline will enable early warning: if apoplastic AsA is low and PN-dependent PTMs are high under a given meteorology, a decline in SIF or a rise in canopy temperature can be interpreted mechanistically rather than phenomenologically (Dopp et al., 2023; Wu et al., 2024; Zou et al., 2024).

A fourth gap is parameter identifiability in the antioxidant and sulfur-detox networks. The ascorbate glutathione cycle, sulfite oxidase/reductase routes, and NADPH supply form a stiff, coupled system; we seldom know which kinetic step limits throughput under a given gas pulse. Metabolic control analysis and Bayesian parameter inference on time-series data generated by step perturbations of O_3_/NO_2_/SO_2_ under controlled light and VPD can rank control coefficients for APX vs CAT vs GR and thereby prescribe where to engineer capacity (enzyme abundance versus substrate pools versus NADPH regeneration). In sulfur detox specifically, we need flux omics that track SO_2_ → HSO_3_^-^/SO_3_²^-^ → SO_4_²^-^/S²^-^ → cysteine → glutathione using stable isotopes (^34^S) to quantify bottlenecks across peroxisome chloroplast–cytosol exchanges (Valero et al., 2015; Li et al., 2018; Xiao et al., 2022; Giampiccolo et al., 2024).

Genetic translation is the fifth gap. The field has identified plausible tolerance levers high apoplastic ascorbate, fast guard-cell kinetics resilient to O_3_-ROS, high-capacity SO/SiR flux, guarded nitrosative PTMs but we lack allele-level blueprints in elite germplasm and a map of trade-offs with growth and pathogen defense. A credible agenda includes allele mining and GWAS across landraces for apoplastic antioxidant capacity and SO and SiR activity, CRISPR base editing of catalytic hotspots in APX/CAT/SOD and SO and SiR, synthetic promoters that boost detox capacity only during pollution episodes (reducing fitness costs), and guard-cell circuit tuning (OST1/SLAC1/RBOH modules) to avoid O_3_-induced sluggishness without sacrificing drought vigilance. Because HO-^1–^derived CO has bona fide signaling roles, targeted edits that preserve developmental benefits while limiting heme-binding toxicity under elevated CO should also be explored. These manipulations must be validated in field-realistic mixture trials, not just chambers (Do et al., 2022; Aarabi et al., 2023; Rovira et al., 2024; Yang et al., 2024).

The sixth gap is phenotyping and data infrastructure. Rapid, scalable, and non-destructive biochemical readouts are needed to calibrate models and screen germplasm. Hyperspectral and thermal imaging from UAVs and towers, together with sun-induced chlorophyll fluorescence, can be benchmarked against leaf chemistry (AsA/GSH pools, PTM panels, sulfite/sulfate ratios) to produce surrogate predictors deployable at scale. Community benchmarks should require a minimal dataset for each trial, pollutant time series, microclimate, $g_{s}$, $A_{n}$, apoplastic pH, sentinel biochemical markers, and harvest traits. A curated, open repository that links these layers across species and climates will accelerate meta-inference and the training of hybrid mechanistic machine-learning models capable of issuing field-level “pollutant injury forecasts (Pizzio et al., 2024; Sadeh et al., 2025).

Agronomic practice and policy form the seventh gap. Irrigation, canopy management, and planting dates can raise or lower stomatal flux at the worst possible hours. Trials that co-optimize water management with pollution risk for example, shifting irrigation to reduce mid-afternoon O_3_ uptake, or using canopy porosity and windbreaks to adjust boundary-layer conductance should be evaluated alongside traditional nutrient and pest programs. On the regulatory side, concentration-only standards cannot encode physiology; adoption of flux- and physiology-aware indices for vegetation (and eventually for SO_2_/NO_2_, not just O_3_) would align air-quality policy with biological dose and recognize mixture penalties explicitly. Future reports should move toward common protocols: co-exposure designs with diurnal light/VPD cycling; real-time $g_{s}$ and leaf temperature, apoplastic pH and redox sensors; explicit reporting of speciation-aware doses (e.g., SAD, PND) alongside conventional metrics; and harmonized endpoints spanning PTMs → organelle function → gas exchange → yield. This standardization will make cross-study synthesis far more powerful, reduce irreproducibility, and highlight truly generalizable tolerance traits (Gámez-Arjona et al., 2022; Chang et al., 2024).

Future investigations should aim to bridge the gap between transcriptomic changes and phenotypic outcomes across a broader range of plant species under realistic field conditions. Integrating high-throughput omics with real-time pollutant exposure data could uncover conserved defense pathways and novel regulatory elements. There is also a need to model pollutant impacts under combined abiotic stresses such as drought or heat, which often co-occur with high atmospheric pollution. Expanding studies beyond Arabidopsis to economically important crops (e.g., wheat, rice, soybean) will enhance translational relevance. Furthermore, cross-disciplinary efforts linking plant physiology, atmospheric modeling, and data-driven AI frameworks will be essential to predict and mitigate pollutant-induced yield losses under future climate scenarios.

## 8 Conclusion

This study synthesized recent advances in understanding the effects of atmospheric pollutants, especially O_3_, NO_2_, SO_2_, and CO, on plant physiological, transcriptomic, and yield related responses. According to multiple studies, O_3_ consistently emerges as a dominant factor in photosynthetic suppression, yield loss, and stomatal regulation. Comparative transcriptomic insights reveal overlapping defense networks but also stressor-specific pathways, underscoring the complexity of plant responses. However, research remains fragmented, with limited integration across species, pollutant types, and environmental contexts. Future work must prioritize multi-stressor frameworks, crop specific validations, and systems level modeling to develop actionable mitigation strategies. This review provides a foundation to inform those next steps. A chemistry centric synthesis reveals that gaseous pollutants differ in upstream reactivity, SO_2_ as a soluble nucleophile that drives sulfitolysis and sulfur detox fluxes, NO_x_ as a nitro- oxidative system that rewires proteins through S-nitrosylation and nitration, O_3_ as an electrophile that primes apoplastic ROS and stomatal dysfunction, and CO as a heme- binding inhibitor with a dose-dependent signaling side but converge on a common physiological cascade, disturbed redox networks, impaired electron transport, altered guard-cell control, and depressed photosynthesis and yield. Comparative evidence is strongest for ozone because physiology-aware exposure metrics connect leaf uptake to national harvests, for NO_x_ and SO_2_, mechanistic clarity at the biochemical level now points to tractable engineering and breeding levers, for CO, the dual role of gas transmitter versus toxin demands careful dose aware interpretation.

Closing the remaining gaps will require field-realistic mixture exposures coupled to diurnal stomatal dynamics, speciation aware dosing, and live redox measurements inside the apoplast, and models that carry biochemical states up to canopy fluxes and economics. With those elements in place, plant science can shift from *post hoc* diagnosis to predictive control, selecting genotypes with resilient antioxidant and sulfur detox capacities, tuning guard cell circuits to avoid O_3_ induced sluggishness without compromising drought response, scheduling irrigation and canopy management to minimize high risk flux windows and informing air quality policy with metrics that reflect biological dose. Because plants are at once victims, sentinels, and mitigators in the atmosphere biosphere exchange, protecting their biochemical integrity safeguards food security, ecosystem services, and public health in a warm, increasingly polluted world.

## Glossary

O_3_: Ozone
NO_x_: Nitrogen Oxides (primarily NO and NO_2_)
SO₂: Sulfur Dioxide
CO: Carbon Monoxide
ROS: Reactive Oxygen Species
RNS: Reactive Nitrogen Species
PTM: Post-Translational Modification
PODy: Phytotoxic Ozone Dose above a threshold y (y often refers to stomatal flux threshold)
PSII: Photosystem II
HO: Heme Oxygenase
SiR: Sulfite Reductase
WRKY/NAC/AP2/ERF: Families of transcription factors (WRKY, NAC, AP2/ERF)
GC–MS: Gas Chromatography–Mass Spectrometry
RNA-seq: RNA Sequencing
ETC: Electron Transport Chain
ABA: Abscisic Acid
Fv/Fm: Maximum Quantum Efficiency of Photosystem II
qPCR: Quantitative Polymerase Chain Reaction
NO₂: Nitrogen Dioxide
PAMP: Pathogen-Associated Molecular Pattern
PRR: Pattern Recognition Receptor
HR: Hypersensitive Response
MAPK: Mitogen-Activated Protein Kinase
JA/SA: Jasmonic Acid/Salicylic Acid
GC: Guard Cell (context-dependent, may also mean Gas Chromatography if separate)

## References

[B1] Aarabi F. Li S. Guo X. Sierla M. Kangasjärvi J. Kangasjärvi S. (2023). Genome-wide association study unveils ascorbate regulation by PAS/LOV PROTEIN during high light acclimation. Plant Physiol. 193, 2037–2054. doi: 10.1093/plphys/kiad323, PMID: 37265123 PMC10602610

[B2] Abdo H. G. Wang C. Jin H. He J. (2022). Coupling the environmental impacts of reactive nitrogen losses and yield responses of staple crops in China. Front. Plant Sci. 13, 927,935. doi: 10.3389/fpls.2022.927935, PMID: 36092406 PMC9450997

[B3] Adaros G. Weigel H. J. Jäger H. J. (1991). Single and interactive effects of low levels of O_3_, SO_2_ and NO_2_ on the growth and yield of spring rape. Environ. pollut. 72, 269–286. doi: 10.1016/0269-7491(91)90002-E, PMID: 15092095

[B4] Agathokleous E. Paoletti E. De Marco A. (2022). isoAOT40: An improved ozone exposure index based on the Annual Ozone Spectrum Profile (AO3SP). J. Forestry Res. 33, 1949–1955. doi: 10.1007/s11676-022-01537-7, PMID: 41810330

[B5] Aguilar-Garrido A. Reyes-Martín M. P. Vidigal P. Abreu M. M. (2023). A green solution for the rehabilitation of marginal lands: The case of *Lablab purpureus* (L.) Sweet grown in technosols. Plants 12, 2682. doi: 10.3390/plants12142682, PMID: 37514296 PMC10385650

[B6] Ahlfors R. Brosché M. Kollist H. Kangasjärvi J. (2009). Nitric oxide modulates ozone-induced cell death, hormone biosynthesis and gene expression in *Arabidopsis thaliana*. Plant J. 58, 1–12. doi: 10.1111/j.1365-313X.2008.03756.x, PMID: 19054359

[B7] Akram N. A. Shafiq F. Ashraf M. (2017). Ascorbic acid-a potential oxidant scavenger and its role in plant development and abiotic stress tolerance. Front. Plant Sci. 8, 613. doi: 10.3389/fpls.2017.00613, PMID: 28491070 PMC5405147

[B8] Antenozio M. L. Caissutti C. Caporusso F. M. Marzi D. Brunetti P. (2024). Urban air pollution and plant tolerance: omics responses to ozone, nitrogen oxides, and particulate matter. Plants 13, 2027. doi: 10.3390/plants13152027, PMID: 39124144 PMC11313721

[B9] Arnaud D. Deeks M. J. Smirnoff N. (2023). RBOHF activates stomatal immunity by modulating both reactive oxygen species and apoplastic pH dynamics in. Arabidopsis. Plant J. 116, 404–415. doi: 10.1111/tpj.16380, PMID: 37421599 PMC10952706

[B10] Astier J. Lindermayr C. (2012). Nitric oxide-dependent posttranslational modification in plants: an update. Int. J. Mol. Sci. 13, 15193–15208. doi: 10.3390/ijms131115193, PMID: 23203119 PMC3509635

[B11] Aziz M. M. Rausch T. Gunes A. (2016). Augmenting sulfur metabolism and herbivore defense in plants: the role of sulfur-containing metabolites. Front. Plant Sci. 7, 458–458. doi: 10.3389/fpls.2016.00458, PMID: 27092166 PMC4824779

[B12] Becana M. Wienkoop S. Matamoros M. A. (2018). Sulfur transport and metabolism in legume root nodules. Front. Plant Sci. 9, 1434. doi: 10.3389/fpls.2018.01434, PMID: 30364181 PMC6192434

[B13] Beckerson D. W. Hofstra G. (1979). Effect of sulphur dioxide and ozone singly or in combination on leaf chlorophyll, RNA, and protein in white bean. Can. J. Bot. 57, 1940–1945. doi: 10.1139/b79-243, PMID: 36563491

[B14] Bloem E. Haneklaus S. Schnug E. (2015). Milestones in plant sulfur research on sulfur-induced resistance (SIR) in Europe. Front. Plant Sci. 5, 779. doi: 10.3389/fpls.2014.00779, PMID: 25642233 PMC4295439

[B15] Brychkova G. (2007). Sulfite oxidase protects plants against sulfur dioxide toxicity. The Plant Journal 50, 696–709. doi: 10.1111/j.1365-313X.2007.03080.x, PMID: 17425719

[B16] Burkhardt J. Hunsche M. (2013). Breath figures” on leaf surfaces—formation and effects of microscopic leaf wetness. Front. Plant Sci. 4, 422. doi: 10.3389/fpls.2013.00422, PMID: 24167510 PMC3807045

[B17] Bussotti F. Pollastrini M. (2017). Observing climate change impacts on European forests: what works and what does not in ongoing long-term monitoring networks. Front. Plant Sci. 8, 629. doi: 10.3389/fpls.2017.00629, PMID: 28487718 PMC5404609

[B18] Chakrabarti B. Pande P. Hayes F. (2024). Application of additional dose of N could sustain rice yield and maintain plant nitrogen under elevated ozone (O_3_) and carbon dioxide (CO_2_) condition. Front. Sustain. Food Syst. 8, 1,477,210. doi: 10.3389/fsufs.2024.1477210, PMID: 41810299

[B19] Chang C.-Y. Guanter L. Frankenberg C. (2024). Coupling sun-induced chlorophyll fluorescence (SIF) with soil–plant–atmosphere research (SPAR) chambers to advance applications of SIF for crop stress research. Remote Sens. Environ. 315, 114462. doi: 10.1016/j.rse.2024.114462, PMID: 41810140

[B20] Chang-Espino M. Fernández I. G. Hayes F. Pleijel H. (2024). Nitrogen modulates the ozone response of Mediterranean wheat: considerations for ozone risk assessment. Sci. Total Environ. 951, 175,718–175,718. doi: 10.1016/j.scitotenv.2024.175718, PMID: 39181251

[B21] Chaparro-Suarez I. G. Meixner F. X. Kesselmeier J. (2011). Nitrogen dioxide (NO_2_) uptake by vegetation controlled by atmospheric concentrations and plant stomatal aperture. Atmospheric Environ. 45, 5742–5750. doi: 10.1016/j.atmosenv.2011.07.021, PMID: 41810140

[B22] Cheesman A. W. (2024). Reduced productivity and carbon drawdown of tropical forests from ground-level ozone exposure. Nature Geoscience 17, 1003–1007. doi: 10.1038/s41561-024-01530-1, PMID: 41803196

[B23] Chen Y. Wang M. Hu L. Liao W. Dawuda M. M. Li C. (2017). Carbon monoxide is involved in hydrogen gas-induced adventitious root development in cucumber under simulated drought stress. Front. Plant Sci. 8, 128. doi: 10.3389/fpls.2017.00128, PMID: 28223992 PMC5293791

[B24] Chmielowska-Bąk J. Jagna E. Sobieszczuk-Nowicka E. M. (2025). Nitro-oxidative nucleotide modifications in plants and associated microorganisms – signalling sensors or stress symptoms. J. Exp. Bot. 76, 3793–3808. doi: 10.1093/jxb/eraf188, PMID: 40322817 PMC12404729

[B25] Clifton O. E. Fiore A. M. Munger J. W. Malyshev S. L. Horowitz L. W. Shevliakova E. . (2020). Dry deposition of ozone over land: processes, measurement, and modeling. Rev. Geophysics 58, e2019RG000670. doi: 10.1029/2019RG000670, PMID: 33748825 PMC7970530

[B26] Concas F. Mineraud J. Lagerspetz E. Varjonen S. Liu X. Puolamäki K. . (2021). Low-cost outdoor air quality monitoring and sensor calibration: a survey and critical analysis. ACM Trans. Sensor Networks 17, 1–44. doi: 10.1145/3446005, PMID: 40727313

[B27] Conklin P. L. Foyer C. H. Hancock R. D. Ishikawa T. Smirnoff N. (2024). Ascorbic acid metabolism and functions. J. Exp. Bot. 75, 2599–2603. doi: 10.1093/jxb/erae143, PMID: 38699987 PMC11066792

[B28] Cook J. Yadav D. S. Hayes F. Booth N. Bland S. Pande P. . (2025). Modelling ozone-induced changes in wheat amino acids and protein quality using a process-based crop model. Biogeosciences 22, 1035–1056. doi: 10.5194/bg-22-1035-2025, PMID: 38859159

[B29] Corpas F. J. Barroso J. B. (2013a). Nitro-oxidative stress vs oxidative or nitrosative stress in higher plants. New Phytologist (JSTOR) 199, 633–635. doi: 10.1111/nph.12380, PMID: 23763656

[B30] Corpas F. J. Barroso J. B. (2013b). Protein tyrosine nitration in higher plants grown under natural and stress conditions. Front. Plant Sci. 4, 29. doi: 10.3389/fpls.2013.00029, PMID: 23444154 PMC3580390

[B31] Corpas F. J. Barroso J. B. (2015). Reactive sulfur species (RSS): possible new players in the oxidative metabolism of plant peroxisomes. Front. Plant Sci. 6, 116. doi: 10.3389/fpls.2015.00116, PMID: 25763007 PMC4340208

[B32] Corpas F. J. González-Gordo S. Palma J. M. (2020). Plant peroxisomes: a factory of reactive species. Front. Plant Sci. 11, 853. doi: 10.3389/fpls.2020.00853, PMID: 32719691 PMC7348659

[B33] Costa-Broseta Á. Perea-Resa C. Castillo M. C. Ruíz M. F. Salinas J. León J. (2018). Nitric oxide controls constitutive freezing tolerance in *Arabidopsis* by attenuating the levels of osmoprotectants, stress-related hormones and anthocyanins. Sci. Rep. 8, 9268. doi: 10.1038/s41598-018-27668-8, PMID: 29915353 PMC6006431

[B34] Custer G. F. Dini-Andreote F. (2022). Embracing complexity in ecosystem response to global change. Environ. Sci. Technol. 56, 9832–9834. doi: 10.1021/acs.est.2c02817, PMID: 35749439

[B35] de Bont L. Donnay N. Couturier J. Rouhier N. (2022). Redox regulation of enzymes involved in sulfate assimilation and in the synthesis of sulfur-containing amino acids and glutathione in plants. Front. Plant Sci. 13, 958490. doi: 10.3389/fpls.2022.958490, PMID: 36051294 PMC9426629

[B36] de Lacroix de Lavalette A. Barucq L. Alric J. Rappaport F. Zito F. (2009). Is the redox state of the ci heme of the cytochrome b6f complex dependent on the occupation and structure of the Qi site and vice versa? J. Biol. Chem. 284, 20822–20829. doi: 10.1074/jbc.M109.016709, PMID: 19478086 PMC2742847

[B37] Delaria E. R. Cohen R. C. (2023). Measurements of atmosphere–biosphere exchange of oxidized nitrogen and implications for the chemistry of atmospheric NOx. Accounts Chem. Res. 56, 1720–1730. doi: 10.1021/acs.accounts.3c00090, PMID: 37347962 PMC10324316

[B38] Delaria E. R. Place B. K. Liu A. X. Cohen R. C. (2020). Laboratory measurements of stomatal NO_2_ deposition to native California trees and the role of forests in the NOx cycle. Atmospheric Chem. Phys. 20, 14023–14041. doi: 10.5194/acp-20-14023-2020, PMID: 38859159

[B39] De Marco A. Sicard P. (2019). Why do we still need to derive ozone critical levels for vegetation protection? Int. J. Environ. Sci. Natural Resour. 21, 164–166. doi: 10.19080/IJESNR.2019.21.556073

[B40] Denis M. Richaud P. (1982). Dynamics of carbon monoxide recombination to fully reduced cytochrome c oxidase in plant mitochondria after low-temperature flash photolysis. Biochem. J. 206, 379–385. doi: 10.1042/bj2060379, PMID: 6293465 PMC1158595

[B41] Devi M. J. Reddy V. R. (2018). Transpiration response of cotton to vapor pressure deficit and its relationship with stomatal traits. Front. Plant Sci. 9, 1572. doi: 10.3389/fpls.2018.01572, PMID: 30420866 PMC6218332

[B42] Dewan S. Lakhani A. (2024). Impact of ozone pollution on crop yield, human health, and associated economic costs in the Indo-Gangetic plains. Sci. Total Environ. 945, 173820. doi: 10.1016/j.scitotenv.2024.173820, PMID: 38866147

[B43] Do H. J. Lee J. H. Kang B.-C. (2022). Development of a genome-edited tomato with high ascorbate content during later stage of fruit ripening through mutation of SlAPX4. Front. Plant Sci. 13, 836916. doi: 10.3389/fpls.2022.836916, PMID: 35498670 PMC9039661

[B44] Dopp I. J. Kalacska M. Mackenzie S. A. (2023). Hydrogen peroxide sensor HyPer7 illuminates tissue-specific plastid redox dynamics. Plant Physiol. 193, 217–228. doi: 10.1093/plphys/kiad307, PMID: 37226328 PMC10702466

[B45] Duan J. Zhang S. Liu X. (2019). Response of gas-exchange characteristics and chlorophyll fluorescence to acute sulfur dioxide exposure in landscape plants. Ecotoxicology Environ. Saf. 171, 122–129. doi: 10.1016/j.ecoenv.2018.12.064, PMID: 30597316

[B46] Dumont S. Rivoal J. (2019). Consequences of oxidative stress on plant glycolytic and respiratory metabolism. Front. Plant Sci. 10, 166. doi: 10.3389/fpls.2019.00166, PMID: 30833954 PMC6387960

[B47] Duque L. Poelman E. H. Steffan-Dewenter I. (2021). Plant age at the time of ozone exposure affects flowering patterns, biotic interactions and reproduction of wild mustard. Scientific Reports 11, 23448. doi: 10.1038/s41598-021-02878-9, PMID: 34873217 PMC8648743

[B48] Dvořák P. Krasylenko Y. Zeiner A. Šamaj J. Takáč T. (2021). Signaling toward reactive oxygen species-scavenging enzymes in plants. Front. Plant Sci. 11, 618,835. doi: 10.3389/fpls.2020.618835, PMID: 33597960 PMC7882706

[B49] Emberson L. (2020). Effects of ozone on agriculture, forests and grasslands. Philos. Trans. R. Soc. A 378, 20190327. doi: 10.1098/rsta.2019.0327, PMID: 32981434 PMC7536038

[B50] Emberson L. D. Buker P. Ashmore M. R. Mills G. Jackson L. S. Agrawal M. . (2009). A comparison of North American and Asian exposure–response data for ozone effects on crop yields. Atmospheric Environ. 43, 1945–1953. doi: 10.1016/j.atmosenv.2009.01.005, PMID: 41810140

[B51] Everett G. Tai A. P. K. Sitch S. Emberson L. D. (2025). Ozone pollution may limit the benefits of irrigation to wheat productivity in India. Biogeosciences 22, 4203–4219. doi: 10.5194/bg-22-4203-2025, PMID: 38859159

[B52] Feng Y. (2024) in Effects of elevated ozone on winter wheat and potential genetic and physiological mechanisms, (Bonn, Germany: FAO AGRIS - International System for Agricultural Science and Technology), (PhD dissertation, Rheinische Friedrich-Wilhelms-Universität Bonn). Available online at: https://www.sciencedirect.com/science/article/pii/S2213231713000943 (Accessed November 12, 2025).

[B53] Feng L. Wei L. Liu Y. Ren J. Liao W. (2023). Carbon monoxide/heme oxygenase system in plant: roles in abiotic stress response and crosstalk with other signal molecules. Nitric. Oxide 138–139, 51–63. doi: 10.1016/j.niox.2023.06.005, PMID: 37364740

[B54] Feng Z. Xu Y. Kobayashi K. Dai L. Zhang T. Agathokleous E. . (2022). Ozone pollution threatens the production of major staple crops in East Asia. Nat. Food 3, 47–56. doi: 10.1038/s43016-021-00422-6, PMID: 37118490

[B55] Fioletov V. E. McLinden C. A. Krotkov N. A. Li C. Joiner J. Theys N. . (2016). A global catalogue of large SO_2_ sources and emissions derived from the Ozone Monitoring Instrument. Atmospheric Chem. Phys. 16, 11497–11519. doi: 10.5194/acp-16-11497-2016, PMID: 38859159

[B56] Fu Y. Yao N. Xie L. (2018). Adenosine 5′-phosphosulfate reductase (APR) in plant sulfur assimilation: structure, regulation, and function. Front. Plant Sci. 9, 642–642. doi: 10.1639/0007-2745-123.2.333, PMID: 29881391

[B57] Gámez-Arjona F. M. Sánchez-Rodríguez C. Montesinos J. C. (2022). The root apoplastic pH as an integrator of plant signaling. Front. Plant Sci. 13, 931979. doi: 10.3389/fpls.2022.931979, PMID: 36082302 PMC9448249

[B58] Giampiccolo S. Dondelinger F. Stumpf M. P. H. (2024). Robust parameter estimation and identifiability analysis with hybrid neural ordinary differential equations in computational biology. NPJ Syst. Biol. Appl. 10, 139. doi: 10.1038/s41540-024-00460-3, PMID: 39609454 PMC11604934

[B59] Gigolashvili T. Kopriva S. (2014). Transporters in plant sulfur metabolism. Front. Plant Sci. 5, 442. doi: 10.3389/fpls.2014.00442, PMID: 25250037 PMC4158793

[B60] Gross F. Durner J. Gaupels F. (2013). Nitric oxide, antioxidants and prooxidants in plant defence responses. Front. Plant Sci. 4, 419. doi: 10.3389/fpls.2013.00419, PMID: 24198820 PMC3812536

[B61] Guarin J. R. Jägermeyr J. Ainsworth E. A. Oliveira F. A. A. Asseng S. Boote K. . (2024). Modeling the effects of tropospheric ozone on the growth and yield of global staple crops with DSSAT v4.8.0. Geoscientific Model. Dev. 17, 2547–2567. doi: 10.5194/gmd-17-2547-2024, PMID: 38859159

[B62] Gullner G. Komives T. Király L. Schröder P. (2018). Glutathione S-transferase enzymes in plant–pathogen interactions. Front. Plant Sci. 9, 1,836. doi: 10.3389/fpls.2018.01836, PMID: 30622544 PMC6308375

[B63] Habermann E. Dias de Oliveira E. A. Contin D. R. San Martin J. A. Curtarelli L. Gonzalez-Meler M. A. . (2019). Stomatal development and conductance of a tropical forage legume are regulated by elevated [CO2] under moderate warming. Front. Plant Sci. 10, 609. doi: 10.3389/fpls.2019.00609, PMID: 31214207 PMC6554438

[B64] Hale (1993). An integrated statistical approach to estimating plant responses to sequential and concurrent gaseous pollutants 35–46.

[B65] Harmens H. Hayes F. Mills G. Sharps K. (2019). Can reduced irrigation mitigate ozone impacts on an ozone-sensitive African wheat variety? Plants 8, 220. doi: 10.3390/plants8070220, PMID: 31336902 PMC6681504

[B66] Harmens H. Mills G. the ICP Vegetation Programme (2015). Twenty-eight years of ICP Vegetation: an overview of its activities. Annali di Botanica 5, 31–43. doi: 10.4462/annbotrm-13064

[B67] Hasan M. M. Rahman M. Skalicky M. Alabdallah N. M. Waseem M. Jahan M. S. . (2021). Ozone induced stomatal regulations, MAPK and phytohormone signaling in plants. Int. J. Mol. Sci. 22, 6304. doi: 10.3390/ijms22126304, PMID: 34208343 PMC8231235

[B68] Hasanuzzaman M. Hossain M. A. Teixeira da Silva J. A. Fujita M. (2012). “ Plant response and tolerance to abiotic oxidative stress: antioxidant defense is a key factor,” in Crop stress and its management: perspectives and strategies ( Springer, Dordrecht), 261–315.

[B69] Hayes F. Sharps K. van Caspel W. E. Klimont Z. Heyes C. Fagerli H. (2025). Global efforts addressing methane emissions is a key factor to further reducing ozone-induced yield losses of crops in Europe. Environ. pollut. 382, 126,654–126,654. doi: 10.1016/j.envpol.2025.126654, PMID: 40516674

[B70] He L. Wei J. Wang Y. Shang Q. Liu J. Yin Y. . (2022). Marked impacts of pollution mitigation on crop yields in China. Earth’s Future 10, e2022EF002936. doi: 10.1029/2022EF002936, PMID: 40890438

[B71] Hoshika Y. Katata G. Deushi M. Watanabe M. Koike T. Müller J. (2015). Ozone-induced stomatal sluggishness changes carbon and water balance of temperate deciduous forests. Sci. Rep. 5, 9871. doi: 10.1038/srep09871, PMID: 25943276 PMC4421795

[B72] Hoshika Y. Omasa K. Paoletti E. (2013). Both ozone exposure and soil water stress are able to induce stomatal sluggishness. Environ. Exp. Bot. 88, 19–23. doi: 10.1016/j.envexpbot.2011.12.004, PMID: 41810140

[B73] Hu Y. Fernández V. Ma L. (2014). Nitrate transporters in leaves and their potential roles in foliar uptake of nitrogen dioxide. Front. Plant Sci. 5, 360. doi: 10.3389/fpls.2014.00360, PMID: 25126090 PMC4115617

[B74] Hu C.-H. Wang P.-Q. Zhang P.-P. Nie X.-M. Li B.-B. Tai L. . (2020). NADPH oxidases: the vital performers and center hubs during plant growth and signaling. Cells 9, 437. doi: 10.3390/cells9020437, PMID: 32069961 PMC7072856

[B75] Hu S. Zhang X. Wang J. (2025). Ozone stress during rice growth impedes grain-filling capacity of inferior spikelets but not that of superior spikelets. Agronomy 15, 1,809–1,809. doi: 10.3390/agronomy15081809, PMID: 41725453

[B76] Husen A. (2021). Morpho-Anatomical, Physiological, Biochemical and Molecular Responses of Plants to Air Pollution (Cham: Springer International Publishing), 203–234.

[B77] Inoue S. Kinoshita T. (2017). Blue light regulation of stomatal opening and the plasma membrane H^+^-ATPase. Plant Physiol. 174, 531–538. doi: 10.1104/pp.17.00166, PMID: 28465463 PMC5462062

[B78] Jiao G. Chen L. Li K. Zhu J. Dong X. Zhu X. . (2025). Worsened ozone pollution exacerbates the loss of agricultural production in China. J. Geophysical Research: Atmospheres 130, e2024JD042781. doi: 10.1029/2024JD042781, PMID: 40890438

[B79] Jin Z. Yan D. Zhang Z. Li M. Wang T. Huang X. . (2023). Effects of elevated ozone exposure on regional meteorology and air quality in China through ozone–vegetation coupling. J. Geophysical Research: Atmospheres 128, e2022JD038119. doi: 10.1029/2022JD038119, PMID: 40890438

[B80] Jolliffe J. B. Pilati S. Moser C. Lashbrooke J. G. (2023). Beyond skin-deep: targeting the plant surface for crop improvement. J. Exp. Bot. 74, 6468–6486. doi: 10.1093/jxb/erad321, PMID: 37589495 PMC10662250

[B81] Kanofsky J. R. Sima P. (1991). Singlet oxygen production from the reactions of ozone with biological molecules. J. Biol. Chem. 266, 9039–9042. doi: 10.1016/S0021-9258(18)31548-5 2026612

[B82] Kasten D. Durner J. Gaupels F. (2017). Gas alert: the NO2 pitfall during NO fumigation of plants. Front. Plant Sci. 8, 85. doi: 10.3389/fpls.2017.00085, PMID: 28197162 PMC5281616

[B83] Kaylor S. D. Snell Taylor S. J. Herrick J. D. (2023). Estimates of biomass reductions of ozone sensitive herbaceous plants in California. Sci. Total Environ. 878, 163134. doi: 10.1016/j.scitotenv.2023.163134, PMID: 37001658 PMC10543089

[B84] Ke P. Ge X. Yu C. Yang D. Kang R. Huang Y. . (2025). Field investigation of leaf-level NO_2_ exchange between atmosphere and mature *Pinus massoniana* in a subtropical forest. J. Geophysical Research: Atmospheres 130, e2024JD040905. doi: 10.1029/2024JD040905, PMID: 40890438

[B85] Khan A. M. Clifton O. E. Bash J. O. Bland S. Booth N. Stoy P. C. . (2025). Ozone dry deposition through plant stomata: multi-model comparison with flux observations and the role of water stress as part of AQMEII4 Activity 2. Atmospheric Chem. Phys. 25, 8613–8635. doi: 10.5194/acp-25-8613-2025, PMID: 40949628 PMC12425119

[B86] Kim S.-W. Kim K.-M. Jeong Y. Seo S. Park Y. Kim J. (2023). Changes in surface ozone in South Korea on diurnal to decadal time scale for the period of 2001–2021. Atmospheric Chem. Phys. 23, 12,867–12,886. doi: 10.5194/acp-23-12867-2023, PMID: 38859159

[B87] Koprivova A. Kopriva S. (2014). Molecular mechanisms of regulation of sulfate assimilation in plants. Front. Plant Sci. 5, 470. doi: 10.3389/fpls.2014.00589, PMID: 25400653 PMC4212615

[B88] Kuźniak E. Kopczewski T. (2020). The chloroplast reactive oxygen species–redox system in plant immunity and disease. Front. Plant Sci. 11, 572,686–572,686. doi: 10.3389/fpls.2020.572686, PMID: 33281842 PMC7688986

[B89] Landmeyer J. E. (2012). Introduction to Phytoremediation of Contaminated Groundwater (Dordrecht: Springer), 245–274.

[B90] Lee H.-S. (2025). Impact of heat and ozone stress on rice growth and productivity: interactive and mitigating effects. Sci. Total Environ. 980, 179,471–179,471. doi: 10.1016/j.scitotenv.2025.179471, PMID: 40306080

[B91] Lee J. Park R. J. Lee H. (2023). Long-term changes of rice yield loss estimated with AOT40 and M7 metrics using comprehensive ozone and rice cultivation data over South Korea. Asian J. Atmospheric Environ. 17, 21. doi: 10.1007/s44273-023-00021-w, PMID: 41810330

[B92] Lefohn A. S. Malley C. S. Smith L. Wells B. Hazucha M. Simon H. . (2018). Tropospheric ozone assessment report: Global ozone metrics for climate change, human health, and crop/ecosystem research. Elementa: Sci. Anthropocene 6, 27. doi: 10.1525/elementa.279, PMID: 30345319 PMC6192432

[B93] León J. (2022). Protein tyrosine nitration in plant nitric oxide signaling. Front. Plant Sci. 13, 859,374. doi: 10.3389/fpls.2022.859374, PMID: 35360296 PMC8963475

[B94] Leppälä J. Salemaa M. Julkunen-Tiitto R. Rousi M. (2022). Ozone and nitrogen dioxide regulate similar gene expression responses in Arabidopsis but natural variation in the extent of cell death is likely controlled by different genetic loci. Front. Plant Sci. 13, 994,779. doi: 10.3389/fpls.2022.994779, PMID: 36340361 PMC9627343

[B95] Leung F. Oliver R. Mercado L. Slevin D. Harper A. (2020). Calibrating soybean parameters in JULES 5.0 from the US-Ne2/3 FLUXNET sites and the SoyFACE-O_3_ experiment. Geoscientific Model. Dev. Discussions 2020, 1–29. doi: 10.5194/gmd-13-6201-2020, PMID: 38859159

[B96] Li Z.-G. Li X.-E. Chen H.-Y. (2022). Sulfur dioxide: an emerging signaling molecule in plants. Front. Plant Sci. 13, 891626. doi: 10.3389/fpls.2022.891626, PMID: 35615134 PMC9125217

[B97] Li D. Shindell D. Lu X. Zhang Y. Zhong J. Seltzer K. . (2022). Surface ozone impacts on major crop production in China from 2010 to 2017. Atmospheric Chem. Phys. 22, 1–14. doi: 10.5194/acp-22-2625-2022, PMID: 38859159

[B98] Li B.-B. Sun L. Xing D. (2018). NAD kinases: metabolic targets controlling redox coenzymes and reducing power partitioning in plant stress and development. Front. Plant Sci. 9, 379. doi: 10.3389/fpls.2018.00379, PMID: 29662499 PMC5890153

[B99] Li L. Yi H. (2012). Differential expression of Arabidopsis defense-related genes in response to sulfur dioxide. Chemosphere 87, 718–724. doi: 10.1016/j.chemosphere.2011.12.064, PMID: 22265681

[B100] Li F. Zhou Z. Levis S. Sitch S. Hayes F. Feng Z. . (2024). Quantifying the role of ozone-caused damage to vegetation in the Earth system: a new parameterization scheme for photosynthetic and stomatal responses. Geoscientific Model. Dev. 17, 6173–6193. doi: 10.5194/gmd-17-6173-2024, PMID: 38859159

[B101] Liu X. F. Hou F. Li G. K. Sang N. (2015). Effects of nitrogen dioxide and its acid mist on reactive oxygen species production and antioxidant enzyme activity in *Arabidopsis* plants. J. Environ. Sci. 34, 93–99. doi: 10.1016/j.jes.2015.03.011, PMID: 26257351

[B102] Liu C. Shi K. (2021). A review on methodology in O_3_–NO_x_–VOC sensitivity study. Environ. pollut. 291, 118249. doi: 10.1016/j.envpol.2021.118249, PMID: 34600066

[B103] Liu X. Sun H.-H. Z. Wang H. (2025). Inflated negative impacts of temperature on global agricultural yields due to ozone omission. Earth’s Future 13, 2024–005,072–2024–005,072. doi: 10.1029/2024EF005072, PMID: 40890438

[B104] Liu H. Wang X. Feng Z. Xu Y. (2025). Ozone pollution-induced yield loss of major staple crops in China and effects from COVID-19. J. Environ. Sci. (in press). 157, 804–820. doi: 10.1016/j.jes.2025.02.034, PMID: 40602926

[B105] Majumdar S. (2023). “ Role of sulfur in protection against major environmental stress in plants,” (Tayor & Francis Group: Apple Academic Press), 473–529. Available online at: https://www.taylorfrancis.com/chapters/edit/10.1201/9781003346203-16/role-sulfur-protection-major-environmental-stress-plants-snehalata-majumdar-falguni-barman-alivia-paul-rita-kundu (Accessed November 18, 2025).

[B106] Mansilla N. Racca S. Gras D. E. González D. H. Welchen E. (2018). The complexity of mitochondrial complex IV: an update of cytochrome c oxidase biogenesis in plants. Int. J. Mol. Sci. 19, 662. doi: 10.3390/ijms19030662, PMID: 29495437 PMC5877523

[B107] Mao J. Tai A. P. K. Yung D. H. Y. Yuan T. Chau K. T. Feng Z. (2024). Multidecadal ozone trends in China and implications for human health and crop yields: a hybrid approach combining a chemical transport model and machine learning. Atmospheric Chem. Phys. 24, 345–366. doi: 10.5194/acp-24-345-2024, PMID: 38859159

[B108] Marion A. Loubet B. Personne E. Massad R.-S. Flechard C. R. Stella P. . (2022). Nitrous acid production and uptake by *Zea mays* plants in growth chambers in the presence of nitrogen dioxide. Sci. Total Environ. 806, 150696. doi: 10.1016/j.scitotenv.2021.150696, PMID: 34597576

[B109] Martí M. C. Jiménez A. Sevilla F. (2020). Thioredoxin network in plant mitochondria: cysteine S-posttranslational modifications and stress conditions. Front. Plant Sci. 11, 571288. doi: 10.3389/fpls.2020.571288, PMID: 33072147 PMC7539121

[B110] Martí-Guillén J. M. Pardo-Hernández M. Martínez-Lorente S. E. Almagro L. Rivero R. M. (2022). Redox post-translational modifications and their interplay in plant abiotic stress tolerance. Front. Plant Sci. 13, 1027730. doi: 10.3389/fpls.2022.1027730, PMID: 36388514 PMC9644032

[B111] Martins L. Trujillo-Hernandez J. A. Reichheld J. P. (2018). Thiol based redox signaling in plant nucleus. Front. Plant Sci. 9, 705. doi: 10.3389/fpls.2018.00705, PMID: 29892308 PMC5985474

[B112] Mata-Pérez C. Begara-Morales J. C. Chaki M. Sánchez-Calvo B. Valderrama R. Padilla M. N. . (2016). Protein tyrosine nitration during development and abiotic stress response in plants. Front. Plant Sci. 7, 1699. doi: 10.3389/fpls.2016.01699, PMID: 27895655 PMC5108813

[B113] Mata-Pérez C. Sánchez-Vicente I. Arteaga N. Gómez-Jiménez S. Fuentes-Terrón A. Oulebsir C. S. . (2023). Functions of nitric oxide-mediated post-translational modifications under abiotic stress. Front. Plant Sci. 14, 1158184. doi: 10.3389/fpls.2023.1158184, PMID: 37063215 PMC10101340

[B114] Maurya A. K. Sandalio L. M. (2023). Reactive oxygen species- and nitric oxide-dependent regulation of ion and metal homeostasis in plants. J. Exp. Bot. 74, 5970–5988. doi: 10.1093/jxb/erad349, PMID: 37668424 PMC10575707

[B115] Mazzuca G. M. Ren X. Loughner C. P. Estes M. Crawford J. H. Pickering K. E. . (2016). Ozone production and its sensitivity to NO_x_ and VOCs: results from the DISCOVER-AQ field experiment in the Baltimore–Washington region. Atmospheric Chem. Phys. 16, 14463–14474. doi: 10.5194/acp-16-14463-2016, PMID: 38859159

[B116] Meo B. S. Ayoub (2024). Environmental pollutants particulate matter (PM2.5, PM10), carbon monoxide (CO), nitrogen dioxide (NO2). Journal of King Saud University–Science 36, 103280. doi: 10.1016/j.jksus.2024.103280, PMID: 41810140

[B117] Meyer U. Köllner B. Willenbrink J. Krause G. H. M. (2000). Effects of different ozone exposure regimes on photosynthesis, assimilates and thousand grain weight in spring wheat. Agriculture Ecosyst. Environ. 78, 49–55. doi: 10.1016/S0167-8809(99)00111-5, PMID: 41674266

[B118] Miles G. P. Samuel M. A. Jones A. M. Ellis B. E. (2009). Suppression of MKK5 reduces ozone-induced signal transmission to both MPK3 and MPK6 and confers increased ozone sensitivity in *Arabidopsis thaliana*. Plant Signaling Behav. 4, 687–692. doi: 10.4161/psb.4.8.9298, PMID: 19820329 PMC2801376

[B119] Mills G. Pleijel H. Braun S. Büker P. Bermejo V. Calvo E. . (2011). New stomatal flux-based critical levels for ozone effects on vegetation. Atmospheric Environ. 45, 5064–5068. doi: 10.1016/j.atmosenv.2011.06.009, PMID: 41810140

[B120] Mir B. A. Kumari R. Rakhra G. Parihar P. Singh R. Raju A. D. . (2024). Sulfur assimilation and regulation of abiotic stress via OMICS. Plant Stress 14, 100630. doi: 10.1016/j.stress.2024.100630, PMID: 41810140

[B121] Mishra S. Sharma A. Srivastava A. K. (2024). Ascorbic acid: a metabolite switch for designing stress-smart crops. Crit. Rev. Biotechnol. 44, 1350–1366. doi: 10.1080/07388551.2023.2286428, PMID: 38163756

[B122] Monks P. S. (2015). Tropospheric ozone and its precursors from the urban to the global scale: from air quality to short-lived climate forcer. Atmospheric Chemistry and Physics 15, 8889–8973. doi: 10.5194/acp-15-8889-2015, PMID: 38859159

[B123] Moura B. B. Bolsoni V. P. de Paula M. D. Dias G. M. de Souza S. R. (2022). Ozone impact on emission of biogenic volatile organic compounds in three tropical tree species from the Atlantic Forest remnants in Southeast Brazil. Front. Plant Sci. 13, 879039. doi: 10.3389/fpls.2022.879039, PMID: 35812949 PMC9263830

[B124] Muneer S. (2014). Effect of CO, NOx and SO2 on ROS production, photosynthesis and ascorbate-glutathione pathway to induce *Fragaria × ananassa* as a hyperaccumulator. Redox Biology 2, 91–98. doi: 10.1016/j.redox.2013.12.006, PMID: 25460723 PMC4297940

[B125] Musselman R. C. Massman W. J. (1999). A critical review and analysis of the use of exposure- and flux-based ozone indices for predicting vegetation effects. Atmospheric Environ. 33, 2629–2649. doi: 10.1016/j.atmosenv.2005.10.064, PMID: 41810140

[B126] Nagasawa K. Setoguchi H. Sakaguchi S. (2025). Recent advances in adaptation genomics in fumarole fields: an overlooked extreme environment. Plant Cell Physiol. 66, 496–505. doi: 10.1093/pcp/pcae122, PMID: 39412112

[B127] Naumann M. Hubberten H. M. Watanabe M. Hänsch R. Schöttler M. A. Hoefgen R. (2018). Sulfite reductase co-suppression in tobacco reveals detoxification mechanisms and downstream responses comparable to sulfate starvation. Front. Plant Sci. 9, 1423. doi: 10.3389/fpls.2018.01423, PMID: 30374361 PMC6196246

[B128] Norby R. J. Zak D. R. (2011). Ecological lessons from free-air CO_2_ enrichment (FACE) experiments. Annu. Rev. Ecology Evolution Systematics 42, 181–203. doi: 10.1146/annurev-ecolsys-102209-144647, PMID: 41139587

[B129] Nowroz N. Burney J. A. Tai A. P. K. Nizami A.-S. (2024). Elevated tropospheric ozone and crop production: potential negative effects and future research needs. Front. Plant Sci. 14, 1,244,515–1,244,515. doi: 10.3389/fpls.2023.1244515, PMID: 38264020 PMC10803661

[B130] Nowroz F. Hasanuzzaman M. Siddika A. Parvin K. Garcia Caparros P. Nahar K. . (2024). Elevated tropospheric ozone and crop production: potential negative effects and plant defense mechanisms. Front. Plant Sci. 14, 1244515. doi: 10.3389/fpls.2023.1244515, PMID: 38264020 PMC10803661

[B131] Odubo T. Catherine E. A. Kosoe (2024). Air Pollutants in the Context of One Health: Fundamentals, Sources, and Impacts (Cham: Springer Nature Switzerland), 75–121.

[B132] Ormrod D. P. (1986). “ Gaseous air pollution and horticultural crop production,” in Horticultural reviews, vol. 8. ( Avi Publishing, Westport, CT), 1–42.

[B133] Oshanova D. Kurmanbayeva A. Bekturova A. Soltabayeva A. Nurbekova Z. Standing D. . (2021). Level of sulfite oxidase activity affects sulfur and carbon metabolism in *Arabidopsis*. Front. Plant Sci. 12, 690830. doi: 10.3389/fpls.2021.690830, PMID: 34249061 PMC8264797

[B134] Ossola R. Farmer D. K. (2024). The chemical landscape of leaf surfaces and its interaction with the atmosphere. Chem. Rev. 124, 5764–5794. doi: 10.1021/acs.chemrev.3c00763, PMID: 38652704 PMC11082906

[B135] Padalko V. F. Posnik M. Adamczyk J. (2024). Mitochondrial aconitase and its contribution to the pathogenesis of neurodegenerative diseases. Int. J. Mol. Sci. 25, 9950. doi: 10.3390/ijms25189950, PMID: 39337438 PMC11431987

[B136] Pande P. Agrawal S. B. Agrawal M. (2024). Ozone dose–response relationships for wheat can be derived using photosynthetic-based stomatal conductance models. Agric. For. Meteorology 356, 110150. doi: 10.1016/j.agrformet.2024.110150, PMID: 41810140

[B137] Paoletti E. Grulke N. E. (2010). Ozone exposure and stomatal sluggishness in different plant physiognomic classes. Environ. pollut. 158, 2664–2671. doi: 10.1016/j.envpol.2010.04.024, PMID: 20537773

[B138] Papazian S. Blande J. D. (2020). Dynamics of plant responses to combinations of air pollutants. Plant Biol. 22, 68–83. doi: 10.1111/plb.12953, PMID: 30584692

[B139] Pei J. Tai A. P. K. Fu J. S. Shindell D. (2024). Long-term trajectory of ozone impact on maize and soybean yields in the United States: A 40-year spatio-temporal analysis. Environ. pollut. 344, 123407. doi: 10.1016/j.envpol.2024.123407, PMID: 38244900

[B140] Pellegrini E. (2011). Ozone stress in *Melissa officinalis* plants assessed by photosynthetic function. Environmental and Experimental Botany 73, 94–101. doi: 10.1016/j.envexpbot.2010.10.006, PMID: 41810140

[B141] Petřivalský M. Luhová L. (2020). Nitrated nucleotides: new players in signaling pathways of reactive nitrogen and oxygen species in plants. Front. Plant Sci. 11, 598. doi: 10.3389/fpls.2020.00598, PMID: 32508862 PMC7248558

[B142] Petrov M. Nikolaeva Z. Dimitrov A. (2023). The impact of anthropogenic activity on the global environment. Science. Business. Soc. 8, 59–64. Available online at: https://stumejournals.com/journals/sbs/2023/2/59 (Accessed November 24, 2025).

[B143] Pino M. E. Mudd J. B. Bailey-Serres J. (1995). Ozone-induced alterations in the accumulation of newly synthesized proteins in leaves of maize. Plant Physiol. 108, 777–785. doi: 10.1104/pp.108.2.777, PMID: 12228510 PMC157400

[B144] Pizzio G. A. Mayordomo C. Coego A. Bono M. Sanchez-Olvera M. Martin-Vasquez C. . (2024). Basal ABA signaling balances transpiration and photosynthesis. Physiologia Plantarum 176, e14494. doi: 10.1111/ppl.14494, PMID: 39210540

[B145] Pleijel H. Danielsson H. Broberg M. C. (2022). Benefits of the Phytotoxic Ozone Dose (POD) index in dose–response functions for wheat yield loss. Atmospheric Environ. 268, 118797. doi: 10.1016/j.atmosenv.2021.118797, PMID: 41810140

[B146] Plöchl M. Lyons T. Ollerenshaw J. Barnes J. (2000). Simulating ozone detoxification in the leaf apoplast through the direct reaction with ascorbate. Planta 210, 454–467. doi: 10.1007/PL00008153, PMID: 10750904

[B147] Podgórska A. Burian M. Szal B. (2017). Extra-cellular but extra-ordinarily important for cells: Apoplastic reactive oxygen species metabolism. Front. Plant Sci. 8, 1353. doi: 10.3389/fpls.2017.01353, PMID: 28878783 PMC5572287

[B148] Postiglione A. E. Muday G. K. (2020). The role of ROS homeostasis in ABA-induced guard cell signaling. Front. Plant Sci. 11, 968. doi: 10.3389/fpls.2020.00968, PMID: 32695131 PMC7338657

[B149] Rahantaniaina M.-S. Tuzet A. Mhamdi A. Noctor G. (2013). Missing links in understanding redox signaling via thiol/disulfide modulation: how is glutathione oxidized in plants? Front. Plant Sci. 4, 477. doi: 10.3389/fpls.2013.00477, PMID: 24324478 PMC3838956

[B150] Rak M. Bénit P. Chrétien D. Bouchereau J. Schiff M. El-Khoury R. . (2016). Mitochondrial cytochrome oxidase deficiency. Clin. Sci. 130, 393–407. doi: 10.1042/CS20150707, PMID: 26846578 PMC4948581

[B151] Ramundo A. Lábádia A. Chachaj-Brekiesz A. Janczak J. Vasiliu T. Garcia-Borràs M. . (2024). Multimodal carbon monoxide photorelease from flavonoids. Organic Lett. 26, 708–712. doi: 10.1021/acs.orglett.3c04141, PMID: 38227978 PMC10825817

[B152] Ran L. (2017). A photosynthesis-based two-leaf canopy stomatal conductance model for meteorology and air quality modeling with WRF/CMAQ PX LSM. Journal of Geophysical Research: Atmospheres, Advancing Earth and Space Scienes 122, 1930–1952. doi: 10.1002/2016JD025583, PMID: 30505641 PMC6260954

[B153] Randewig D. Schliesky S. Prokchorchik M. Meyer A. J. Hänsch R. Mendel R. R. (2012). Sulfite oxidase controls sulfur metabolism under SO_2_ exposure in *Arabidopsis thaliana*. Plant Cell Environ. 35, 100–115. doi: 10.1111/j.1365-3040.2011.02420.x, PMID: 21895698

[B154] Ren Z. Wang R.-Y. Huang X.-Y. Wang Y. (2022). Sulfur compounds in regulation of stomatal movement. Front. Plant Sci. 13, 846518. doi: 10.3389/fpls.2022.846518, PMID: 35360293 PMC8963490

[B155] Rovira A. Veciana N. Basté-Miquel A. Quevedo M. Locascio A. Yenush L. . (2024). PIF transcriptional regulators are required for rhythmic stomatal movements. Nat. Commun. 15, 4540. doi: 10.1038/s41467-024-48669-4, PMID: 38811542 PMC11137129

[B156] Sadeh R. Alchanatis V. Ben-David R. Peleg Z. Herrmann I. (2025). UAV-borne hyperspectral and thermal imagery integration empowers genetic dissection of wheat stomatal conductance. Comput. Electron. Agric. 235, 110411. doi: 10.1016/j.compag.2025.110411, PMID: 41810140

[B157] Salguero-Linares J. Serrano I. Ruiz-Solani N. Salas-Gómez M. Phukan U. J. González V. M. . (2022). Robust transcriptional indicators of immune cell death revealed by spatiotemporal transcriptome analyses. Mol. Plant 15, 1059–1075. doi: 10.1016/j.molp.2022.04.010, PMID: 35502144

[B158] Salzmann M. Ming Y. Golaz J.-C. Ginoux P. A. Morrison H. Gettelman A. . (2010). Two-moment bulk stratiform cloud microphysics in the GFDL AM3 GCM: description, evaluation, and sensitivity tests. Atmospheric Chem. Phys. 10, 8037–8064. doi: 10.5194/acp-10-8037-2010, PMID: 38859159

[B159] Schiferl L. D. Shah V. Payne V. H. Worden H. M. Cady-Pereira K. E. Jiang Z. (2024). Multi-year observations of variable incomplete combustion in the New York megacity. Atmospheric Chem. Phys. 24, 10129–10142. doi: 10.5194/acp-24-10129-2024, PMID: 38859159

[B160] Schucht S. (2021). “ Wheat yield loss in 2019 in Europe due to ozone exposure,” in Eionet Report – ETC/ATNI 2021/17. (Kjeller: European Topic Centre on Air pollution, transport, noise and industrial pollution).

[B161] Scuffi D. Lamattina L. Garcia-Mata C. (2016). Gasotransmitters and stomatal closure: is there redundancy, concerted action, or both? Front. Plant Sci. 7, 277. doi: 10.3389/fpls.2016.00277, PMID: 27014301 PMC4791407

[B162] Sedlářová M. Luhová L. (2025). Progress in plant nitric oxide studies: implications for phytopathology and plant protection. Int. J. Mol. Sci. 26, 2087. doi: 10.3390/ijms26052087, PMID: 40076711 PMC11899914

[B163] Shang B. He L. Bao M. Li Y. Xu Y. Shao Z. . (2024). Effects of elevated ozone on physiology, growth, yield and grain quality of rice (*Oryza sativa* L.): an ozone gradient experiment. Agriculture Ecosyst. Environ. 363, 108858. doi: 10.1016/j.agee.2023.108858, PMID: 41810140

[B164] Sharma U. Bekturova A. Ventura Y. Sagi M. (2020). Sulfite oxidase activity level determines the sulfite toxicity effect in leaves and fruits of tomato plants. Agronomy 10, 694–694. doi: 10.3390/agronomy10050694, PMID: 41725453

[B165] Singh P. Lamabam (2012), 133–163.

[B166] Singhal R. K. Saha D. Skalicky M. Mishra U. N. Chauhan J. Behera L. P. . (2021). Crucial cell signaling compounds crosstalk and integrative multi-omics techniques for salinity stress tolerance in plants. Front. Plant Sci. 12, 670369. doi: 10.3389/fpls.2021.670369, PMID: 34484254 PMC8414894

[B167] Slot M. Slot M. W. Winter K. Way D. A. (2024). The stomatal response to vapor pressure deficit drives the apparent temperature response of photosynthesis in tropical forests. New Phytol. 244, 1238–1249. doi: 10.1111/nph.19806, PMID: 38736030

[B168] Sowa S. Roos E. E. Caughey W. S. (1993). Effector molecules to probe cytochrome c oxidase activity in germinating *Phaseolus vulgaris* L. seeds. J. Plant Physiol. 141, 647–653. doi: 10.1016/S0176-1617(11)81568-3, PMID: 41799633

[B169] Staszek P. Gniazdowska A. (2020). Peroxynitrite induced signaling pathways in plant response to non-proteinogenic amino acids. Planta 252, 5. doi: 10.1007/s00425-020-03411-4, PMID: 32535658 PMC7293691

[B170] Stocker B. D. Zscheischler J. Keenan T. F. Prentice I. C. Seneviratne S. I. Peñuelas J. . (2018). Quantifying soil moisture impacts on light use efficiency across biomes. New Phytol. 218, 1430–1449. doi: 10.1111/nph.15123, PMID: 29604221 PMC5969272

[B171] Takahashi M. Arimura G.-I. Morikawa H. (2019). Dual nitrogen species involved in foliar uptake of nitrogen dioxide in *Arabidopsis thaliana*. Plant Signaling Behav. 14, e1582263. doi: 10.1080/15592324.2019.1582263, PMID: 30810449 PMC6512919

[B172] Takahashi Y. Bosmans K. C. Hsu P.-K. Paul K. Seitz C. Yeh C.-Y. . (2022). Stomatal CO_2_/bicarbonate sensor consists of two interacting protein kinases, Raf-like HT1 and non-kinase-activity requiring MPK12/MPK4. Sci. Adv. 8, eabq6161. doi: 10.1126/sciadv.abq6161, PMID: 36475789 PMC9728965

[B173] Tiew T. W. Y. Sheahan M. B. Rose R. J. (2015). Peroxisomes contribute to reactive oxygen species homeostasis and cell division induction in Arabidopsis protoplasts. Front. Plant Sci. 6, 658. doi: 10.3389/fpls.2015.00658, PMID: 26379686 PMC4549554

[B174] Tiwari S. Agrawal M. Marshall F. M. (2006). Evaluation of ambient air pollution impact on carrot plants at a suburban site using open top chambers. Environ. Monit. Assess. 119, 15–30. doi: 10.1007/s10661-005-9001-z, PMID: 16736274

[B175] Torsethaugen G. Pell E. J. Assmann S. M. (1999). Ozone inhibits guard cell K+ channels implicated in stomatal opening. Proc. Natl. Acad. Sci. United States America 96, 13577–13582. doi: 10.1073/pnas.96.23.13577, PMID: 10557363 PMC23990

[B176] Tripathi D. K. Nam H. G. Oldenburg D. J. Bendich A. J. (2020). Reactive oxygen species, antioxidant agents, and DNA damage in developing maize mitochondria and plastids. Front. Plant Sci. 11, 596. doi: 10.3389/fpls.2020.00596, PMID: 32508860 PMC7248337

[B177] Turc B. Vollenweider P. Le Thiec D. Gandin A. Schaub M. Cabané M. . (2021). Dynamics of foliar responses to O3 stress as a function of phytotoxic O3 dose in hybrid poplar. Front. Plant Sci. 12, 679852. doi: 10.3389/fpls.2021.679852, PMID: 34262582 PMC8273248

[B178] Ugalde J. M. Fuchs P. Nietzel T. Cutolo E. A. Vothknecht U. C. Holuigue L. . (2021). The latest HyPe(r) in plant H_2_O_2_ biosensing. Plant Physiol. 187, 480–484. doi: 10.1093/plphys/kiab306, PMID: 34608965 PMC8491017

[B179] Upadhayay V. K. Jaiswal A. Kumari G. (2024). “ Role of nano-bioinoculants in alleviation of abiotic stresses for sustainable agriculture,” in Challenges in the green world: abiotic stress and plants ( Elite Publishing House), 61–85.

[B180] Vahisalu T. Puzõrjova I. Brosché M. Valk E. Lepiku M. Moldau H. . (2010). Ozone-triggered rapid stomatal response involves the production of reactive oxygen species and is controlled by SLAC1 and OST1. Plant J. 62, 442–453. doi: 10.1111/j.1365-313X.2010.04159.x, PMID: 20128877

[B181] Vainonen J. P. Kangasjärvi J. (2015). Plant signalling in acute ozone exposure. Plant Cell Environ. 38, 240–252. doi: 10.1111/pce.12273, PMID: 24417414

[B182] Valero E. Serrano-Andrés L. García-Carmona F. (2015). Modeling the ascorbate–glutathione cycle in chloroplasts under light/dark conditions. BMC Syst. Biol. 9, 11. doi: 10.1186/s12918-015-0239-y, PMID: 26797294 PMC4722729

[B183] Vazquez Santiago J. Hata H. Martinez-Noriega E. J. Inoue K. (2024). Ozone trends and their sensitivity in global megacities under the warming climate. Nat. Commun. 15, 10236. doi: 10.1038/s41467-024-54490-w, PMID: 39592683 PMC11599728

[B184] Ventura I. Brunetti P. Panzeri D. De Paolis A. Torti S. Rampino P. . (2020). *Arabidopsis* phenotyping reveals the importance of alcohol dehydrogenase and pyruvate decarboxylase for aerobic plant growth. Sci. Rep. 10, 16669. doi: 10.1038/s41598-020-73704-x, PMID: 33028901 PMC7542448

[B185] Wang F. Feng Z. Shang B. Li Y. He L. Shang Y. (2024). Estimation of surface ozone effects on winter wheat yield across the North China Plain. Agronomy 14, 2326. doi: 10.3390/agronomy14102326, PMID: 41725453

[B186] Wang M. Jia Y. Xu Z. Xia Z. (2016). Impairment of sulfite reductase decreases oxidative stress tolerance in *Arabidopsis thaliana*. Front. Plant Sci. 7, 1843. doi: 10.3389/fpls.2016.01843, PMID: 27994615 PMC5133253

[B187] Wang J. Kong W. Li H. Sun X. Sun Y. Liu Y. (2025). Effects of meteorological factors on the retention of particulate matter in lawn grass blades. Front. Plant Sci. 16, 1,495,212. doi: 10.3389/fpls.2025.1495212, PMID: 39912097 PMC11794498

[B188] Wang M. Liao W. (2016). Carbon monoxide as a signaling molecule in plants. Front. Plant Sci. 7, 572. doi: 10.3389/fpls.2016.00572, PMID: 27200045 PMC4850744

[B189] Wang Y. Wang J. Li Z. Song J. Liu Y. Qiu Y. . (2024). Atmospheric NO_2_ enhances tolerance to low temperature by promoting nitrogen and carbon metabolism in tobacco. Environ. Exp. Bot. 225, 105860. doi: 10.1016/j.envexpbot.2024.105860, PMID: 41810140

[B190] Wei X. Lyu S. Yu Y. Wang Z. Liu H. Pan D. . (2017). Phylloremediation of air pollutants: exploiting the potential of plant leaves and leaf-associated microbes. Front. Plant Sci. 8, 1318. doi: 10.3389/fpls.2017.01318, PMID: 28804491 PMC5532450

[B191] Welle M. Niether W. Stöhr C. (2024). The underestimated role of plant root nitric oxide emission under low-oxygen stress. Front. Plant Sci. 15, 1290700. doi: 10.3389/fpls.2024.1290700, PMID: 38379951 PMC10876902

[B192] Weng X. Zhu L. Yu S. Liu Y. Ru Y. Zhang Z. . (2022). Carbon monoxide promotes stomatal initiation by regulating the expression of two EPF genes in *Arabidopsis* cotyledons. Front. Plant Sci. 13, 1029703. doi: 10.3389/fpls.2022.1029703, PMID: 36438138 PMC9691970

[B193] Wikström M. Krab K. Sharma V. (2018). Oxygen activation and energy conservation by cytochrome c oxidase. Chem. Rev. 118, 2469–2490. doi: 10.1021/acs.chemrev.7b00664, PMID: 29350917 PMC6203177

[B194] Wu G. Peng B. Guan K. Ainsworth E. A. Bernacchi C. J. (2024). Solar-induced chlorophyll fluorescence captures the effects of elevated ozone on canopy structure and acceleration of senescence in soybean. J. Exp. Bot. 75, 350–363. doi: 10.1093/jxb/erad356, PMID: 37702411

[B195] Xia Z. Wang M. Xu Z. (2018). the maize sulfite reductase is involved in cold and oxidative stress responses. Front. Plant Sci. 9, 1,680. doi: 10.3389/fpls.2018.01680, PMID: 30498506 PMC6249382

[B196] Xiao W. Li H. Anjum N. A. (2021). The multiple roles of ascorbate in the abiotic stress response of plants: antioxidant, cofactor, and regulator. Front. Plant Sci. 12, 598,173. doi: 10.3389/fpls.2021.598173, PMID: 33912200 PMC8072462

[B197] Xiao Z. Lu Y. Zou Y. Zhang C. Ding L. Luo K. . (2022). Gene Identification, expression analysis and molecular docking of ATP sulfurylase in the selenization pathway of Cardamine hupingshanensis. BMC Plant Biol. 22, 491. doi: 10.1186/s12870-022-03872-7, PMID: 36253724 PMC9578213

[B198] Xie Y. Hu X. (2018). Hydrogen sulfide-induced tolerance to abiotic stress in plants: physiological and molecular mechanisms. Front. Plant Sci. 9, 286–286. 29706971

[B199] Xu E. Tikkanen M. Seyednasrollah F. Kangasjärvi S. Brosché M. (2022). Simultaneous ozone and high light treatments reveal an important role for the chloroplast in co-ordination of defense signaling. Front. Plant Sci. 13, 883002. doi: 10.3389/fpls.2022.883002, PMID: 35873979 PMC9303991

[B200] Xuan L. Li J. Wang X. Wang C. (2020). Crosstalk between hydrogen sulfide and other signal molecules regulates plant growth and development. Int. J. Mol. Sci. 21, 4593. doi: 10.3390/ijms21134593, PMID: 32605208 PMC7370202

[B201] Yadav D. Singh S. B. Agrawal M. (2021). Ozone flux–effect relationship for early and late-sown Indian wheat cultivars: growth, biomass, and yield. Field Crops Res. 263, 108076. doi: 10.1016/j.fcr.2021.108076, PMID: 41810140

[B202] Yang Y. Li J. Zhang S. Sun Y. (2024). Fluorescent probes for sensing peroxynitrite: biological applications. Redox Rep. 29, 2430157. doi: 10.1080/13510002.2024.2430157, PMID: 39581574 PMC11587728

[B203] Yang Y. Shan J. Xia Y. Gui Y. (2024). Fluorescent probes for sensing peroxynitrite: Biological applications. Redox Report. 29, 2430157. 39581574 10.1080/13510002.2024.2430157 PMC11587728

[B204] Yang LiMing Y. L. Ji JianHui J. J. Wang HongLiang W. H. Harris-Shultz K. R. Abd_allah E. F. Luo YuMing L. Y. . (2016). Carbon monoxide interacts with auxin and nitric oxide to cope with iron deficiency in Arabidopsis. Frontiers in Plant Science(CABAI Digital Library) Vol. 7. doi: 10.3389/fpls.2016.00112, PMID: 27014280 PMC4780267

[B205] Yarmolinsky D. Brychkova G. Fluhr R. Sagi M. (2013). Sulfite reductase protects plants against sulfite toxicity. Plant Physiol. 161, 725–743. doi: 10.1104/pp.112.207712, PMID: 23221833 PMC3561015

[B206] Yonekura-Sakakibara K. Onda Y. Ashikari T. Tanaka Y. Kusumi T. Hase T. . (2000). Analysis of reductant supply systems for ferredoxin-dependent sulfite reductase in photosynthetic and nonphotosynthetic organs of maize. Plant Physiol. 122, 887–894. doi: 10.1104/pp.122.3.887, PMID: 10712553 PMC58925

[B207] Yue W. Wang J. Zhang H. Guo D. He G. Sun G. (2021). A intermediate concentration of atmospheric nitrogen dioxide enhances PSII activity and inhibits PSI activity in expanded leaves of tobacco seedlings. Ecotoxicology Environ. Saf. 209, 111844. doi: 10.1016/j.ecoenv.2020.111844, PMID: 33383337

[B208] Zhang W. Cheng Y. Maksym R. Chen X. Zhou Y. Tan X.-L. (2025). The glycosylation status of small molecules impacts different aspects of plant immunity. Physiologia Plantarum 177, e70292. doi: 10.1111/ppl.70292, PMID: 40432173

[B209] Zhang W. Feng Z. Li J. Xie Y. Xu Y. (2017). Quantification of ozone exposure– and stomatal uptake–yield response relationships for soybean in Northeast China. Sci. Total Environ. 599–600, 710–720. doi: 10.1016/j.scitotenv.2017.04.231, PMID: 28494296

[B210] Zhang H. Whitelegge J. P. Cramer W. A. (2004). Characterization of the high-spin heme x in the cytochrome b_6_f complex of oxygenic photosynthesis. Biochemistry 43, 16329–16336. doi: 10.1021/bi048363p, PMID: 15610027

[B211] Zhang J. Ghirardo A. Gori A. Albert A. Buegger F. Pace R. . (2020). Improving air quality by nitric oxide consumption of climate-resilient trees suitable for urban greening. Front. Plant Sci. 11, 549,913. doi: 10.3389/fpls.2020.549913, PMID: 33117411 PMC7550725

[B212] Zheng B. Chevallier F. Yin Y. Ciais P. Fortems-Cheiney A. Deeter M. N. . (2019). Global atmospheric carbon monoxide budget 2000–2017 inferred from multi-species atmospheric inversions. Earth System Sci. Data 11, 1411–1436. doi: 10.5194/essd-11-1411-2019, PMID: 38859159

[B213] Zhu J. Tai A. P. K. Yim S. H. L. (2022). Effects of ozone–vegetation interactions on meteorology and air quality in China using a two-way coupled land–atmosphere model. Atmospheric Chem. Phys. 22, 765–782. doi: 10.5194/acp-22-765-2022, PMID: 38859159

[B214] Zhu X. Dong H. Huang Y. Ren W. (2025). Assessing ozone pollution and climate change impacts on winter wheat: flux modeling vs. dose-response modeling. J. Environ. Manage. 387, 125,767–125,767. doi: 10.1016/j.jenvman.2025.125767, PMID: 40398280

[B215] Zou C. Joiner J. Guanter L. Köhler P. Sun Y. (2024). TCSIF: a temporally consistent global GOME-2A solar-induced chlorophyll fluorescence dataset with correction for sensor degradation. Earth System Sci. Data 16, 2789–2809. doi: 10.5194/essd-16-2789-2024, PMID: 38859159

